# A photoswitchable fluorescent protein for hours-time-lapse and sub-second-resolved super-resolution imaging

**DOI:** 10.1093/jmicro/dfab001

**Published:** 2021-01-22

**Authors:** Tetsuichi Wazawa, Ryohei Noma, Shusaku Uto, Kazunori Sugiura, Takashi Washio, Takeharu Nagai

**Affiliations:** The Institute of Scientific and Industrial Research (SANKEN), Osaka University, 8-1 Mihogaoka, Ibaraki, Osaka 567-0047, Japan; The Institute of Scientific and Industrial Research (SANKEN), Osaka University, 8-1 Mihogaoka, Ibaraki, Osaka 567-0047, Japan; The Institute of Scientific and Industrial Research (SANKEN), Osaka University, 8-1 Mihogaoka, Ibaraki, Osaka 567-0047, Japan; The Institute of Scientific and Industrial Research (SANKEN), Osaka University, 8-1 Mihogaoka, Ibaraki, Osaka 567-0047, Japan; The Institute of Scientific and Industrial Research (SANKEN), Osaka University, 8-1 Mihogaoka, Ibaraki, Osaka 567-0047, Japan; Transdimensional Life Imaging Division, Institute for Open and Transdisciplinary Research Initiatives, Osaka University, 2-1 Yamadaoka, Suita, Osaka 565-0871, Japan; The Institute of Scientific and Industrial Research (SANKEN), Osaka University, 8-1 Mihogaoka, Ibaraki, Osaka 567-0047, Japan; Transdimensional Life Imaging Division, Institute for Open and Transdisciplinary Research Initiatives, Osaka University, 2-1 Yamadaoka, Suita, Osaka 565-0871, Japan

**Keywords:** super-resolution microscopy, reversibly photoswitchable fluorescent protein, biocompatibility, chromophore phenolate, cell dynamics, multiple equilibria

## Abstract

Reversibly photoswitchable fluorescent proteins (RSFPs) are a class of fluorescent proteins whose fluorescence can be turned on and off by light irradiation. RSFPs have become essential tools for super-resolution (SR) imaging. Because most SR imaging techniques require high-power-density illumination, mitigating phototoxicity in cells due to intense light irradiation has been a challenge. Although we previously developed an RSFP named Kohinoor to achieve SR imaging with low phototoxicity, the photoproperties were insufficient to move a step further to explore the cellular dynamics by SR imaging. Here, we show an improved version of RSFP, Kohinoor2.0, which is suitable for SR imaging of cellular processes. Kohinoor2.0 shows a 2.6-fold higher fluorescence intensity, 2.5-fold faster chromophore maturation and 1.5-fold faster off-switching than Kohinoor. The analysis of the pH dependence of the visible absorption band revealed that Kohinoor2.0 and Kohinoor were in equilibria among multiple fluorescently bright and dark states, with the mutations introduced into Kohinoor2.0 bringing about a higher stabilization of the fluorescently bright states compared to Kohinoor. Using Kohinoor2.0 with our SR imaging technique, super-resolution polarization demodulation/on-state polarization angle narrowing, we conducted 4-h time-lapse SR imaging of an actin filament network in mammalian cells with a total acquisition time of 480 s without a noticeable indication of phototoxicity. Furthermore, we demonstrated the SR imaging of mitochondria dynamics at a time resolution of 0.5 s, in which the fusion and fission processes were clearly visualized. Thus, Kohinoor2.0 is shown to be an invaluable RSFP for the SR imaging of cellular dynamics.

## Introduction

Reversibly photoswitchable fluorescent proteins (RSFPs) are a class of fluorescent proteins whose fluorescence states can be controlled reversibly and cyclically by light irradiation [1]. Making use of the reversible photoswitching nature, RSFPs have been extensively exploited as fluorescent probes for super-resolution (SR) bioimaging, as well as in applications including protein movement tracking, subcellular environment sensing and the optical control of protein activity [1–3]. If an RSFP converts into a fluorescently dark state (off state) with the irradiation of a wavelength of light, this transition is called off-switching. If the off state of RSFP converts into a fluorescently bright state (on state) with the irradiation of another wavelength of light, then the transition is called on-switching. These photoswitching processes are known to involve the interplay of the chromophore isomerization inside the β-barrel and the protonation/deprotonation of the phenolic group in the chromophore [4].

SR microscopy is a technique to perform observation with a fluorescence microscope at a higher spatial resolution than that with conventional optical microscopy, in which the spatial resolution is limited by the diffraction limit of light [5]. In the linear fluorescence regime, the intensity of the fluorescence signal from fluorescent molecules is usually assumed to be proportional to the power density of the excitation light. In contrast, some SR microscopy methods manipulate fluorescent molecules with light to induce nonlinear fluorescence responses and circumvent the diffraction limit, which is often performed by using RSPFs.

Super-resolution polarization demodulation/excitation polarization angle narrowing (SPoD-ExPAN) is an SR microscopy method used to resolve fluorescent molecules with respect to orientation, resolving fine structures made of well-oriented fluorescent molecules [6]. SPoD-ExPAN used linearly polarized lights for excitation and stimulated emission depletion. Although the light for the stimulated emission depletion improves the spatial resolution, its power density can be as intense as 1.7 MW cm^−2^ [6]. Unfortunately, irradiation at such a high-power density often gives rise to phototoxicity in live cells, hampering the long-term observation of dynamic cellular processes. To circumvent this issue, we reorganized SPoD-ExPAN by exploiting the photoswitching of an RSFP, Kohinoor [7], instead of using the stimulated emission depletion mechanism, and successfully achieved highly biocompatible SR observation at an illumination power density of $\sim$1 W cm^−2^, representing a decrease in the magnitude of the power density by 6 orders [8]. We denoted this SR technique as super-resolution polarization demodulation/on-state polarization angle narrowing (SPoD-OnSPAN) [9]. RSFPs have also been shown to be useful for other SR bioimaging methods, such as reversible saturable/switchable optical fluorescence transitions (RESOLFT) [7,10–12] and nonlinear structured illumination microscopy (NL-SIM) [13,14].

Kohinoor is an RSFP designed for SR imaging with a very low phototoxicity [7]. It undergoes off-switching with irradiation at 405 nm and both on-switching and fluorescence excitation at 488 nm. As Kohinoor shares the same wavelength for fluorescence excitation and on-switching, it is denoted as a positively reversibly photoswitchable fluorescent protein (pRSFP). Although Kohinoor has allowed for SR observation by RESOLFT, NL-SIM and SPoD-OnSPAN with very low doses of light [7–9,14], some photoproperties, such as fluorescence intensity and photoswitching speed, have yet to be improved for time-lapse and high-time-resolution SR imaging.

In this study, we developed a novel pRSFP, Kohinoor2.0, which exhibits a higher fluorescence intensity, a faster off-switching rate and a faster maturation rate than Kohinoor. These improved properties are suitable for time-lapse and high-time-resolution SR imaging. To determine how the fluorescence intensity was enhanced in Kohinoor2.0, we analyzed the protonation state of the phenolic group in the chromophore to evaluate its multiple equilibria in relation to fluorescence intensity. Furthermore, using Kohinoor2.0, we demonstrated hours-time-lapse SR imaging of actin filament network and sub-second-resolved SR imaging of mitochondria in mammalian cells.

## Materials and methods

### Construction of expression vectors

We amplified the genes of Kohinoor and Kohinoor2.0 by polymerase chain reaction (PCR) to subclone them into the bacterial expression vector pRSET_B_ (Invitrogen, Carlsbad, CA, USA) between the BamHI and EcoRI restriction enzyme sites. We used the pRSET_B_-Kohinoor vector for error-prone PCR random mutagenesis. For the maturation kinetics study, we ligated the Kohinoor2.0 gene into the pBAD vector (Thermo Fisher Scientific, Waltham, MA, USA) between the BglII and HindIII restriction sites. For microscopy observation, we constructed pcDNA3 mammalian expression vectors containing genes of Kohinoor2.0 or Kohinoor fused with histone 2B, a targeting sequence of subunit-VIII precursor of human cytochrome *c* oxidase (tsCOX8) [15], β-tubulin, LifeAct [16], vimentin, clathrin, fibrillarin and zyxin. We also constructed expression vectors containing genes of mCherry [17] connected to Kohinoor2.0 through P2A [18] (mCherry-P2A-Kohinoor2.0) and mCherry connected to Kohinoor through P2A (mCherry-P2A-Kohinoor).

### Mutagenesis and other cloning reaction

We performed error-prone PCR amplifications using Taq polymerase (Takara Bio, Kusatsu, Japan) and DNA shuffling by the staggered extension process (StEP) [19] to derive a mutant of Kohinoor-M40V/L153V/S162L/R170L/L185M/Y188N/E214V (Kohinoor2.0, see Development of Kohinoor2.0 in Results and discussion). We used KOD-Plus DNA polymerase (Toyobo, Osaka, Japan) for non-mutagenic PCR amplification. We determined the complementary DNA (cDNA) sequences by dye-terminator cycle sequencing using the BigDye Terminator v1.1 Cycle Sequencing Kit and an ABI PRISM Genetic Analyzer (Thermo Fisher Scientific).

### Screening of photoswitchable fluorescent protein mutants

We spread *Escherichia coli* JM109 (DE3) cells transformed with the DNAs of mutant Kohinoor library on 95-mm LB agar plates and incubated them at 37°C for 16 h. We screened mutants with a high brightness and fast photoswitching by using an in-house-built illumination system [7]. We irradiated the Kohinoor mutants expressed in the *E. coli* cells with an excitation light at 475 nm from an LED light source (SPECTRA X Light Engine; Lumencor, Beaverton, OR, USA) and took the fluorescence through a bandpass filter (center wavelength, 525 nm; FF01-525/45; Semrock, Rochester, NY, USA) with a charge-coupled device (CCD) camera (01-QIClick-F-M12; QImaging, Surrey, BC, Canada). We measured the photoswitching with irradiation at 475 and 386 nm for on-switching and off-switching, respectively. We analyzed the data by ImageJ-Fiji software [20].

### Protein expression and purification

We expressed Kohinoor2.0 and Kohinoor with an N-terminal polyhistidine tag in *E. coli* JM109 (DE3) at 23°C for 65 h in LB medium supplemented with 0.1 mg ml^−1^ carbenicillin. We collected the cells and ruptured them with a French press (French Press G-M Model 11; Glen Mills, Clifton, NJ, USA). We purified the recombinant proteins from the supernatant by affinity chromatography with a Ni-NTA agarose (QIAGEN, Hilden, Germany) column followed by gel filtration on a PD-10 column (GE Healthcare Bio-Sciences, Pittsburgh, PA, USA) equilibrated with 20 mM HEPES-NaOH (pH 7.4).

### Protein characterization

We used a U-3900 spectrophotometer (Hitachi High-Tech Science, Tokyo, Japan), an F-7000 fluorescence spectrophotometer (Hitachi High-Tech Science) and a Quantarus-QY spectrophotometer (C11347-01; Hamamatsu Photonics, Hamamatsu, Japan) to measure the absorption spectrum, fluorescence spectrum and absolute fluorescence quantum yield, respectively, of a protein in a cuvette. Before the spectrum measurement, we performed on-switching or off-switching in order to examine a fluorescent protein in either state. For on-switching, we irradiated a protein solution in a cuvette with an on-switching light (center wavelength, 475; band width, 28 nm) from SPECTRA X Light Engine at a power density of 0.69 mW cm^−2^ for 10 min. For off-switching, we irradiated a protein solution with an off-switching light (center wavelength, 386 nm; band width, 24 nm) at a power density of 0.69 mW cm^−2^ for 10 min. After on- or off-switching, we immediately performed spectrum measurement. To determine the molar extinction coefficients of Kohinoor2.0 and Kohinoor, we conducted the alkali denaturation method with a molar extinction coefficient of 44 000 M^−1^ cm^−1^ at 447 nm for the chromophore [21].

We measured the pH-dependent absorption spectra of Kohinoor2.0 and Kohinoor in the on state. We mixed 490 µl of buffer solution and 10 µl of a fluorescent protein solution, performed on-switching (see the paragraph above) and then measured the absorption spectrum. For a pH from 5 to 9, we prepared an aqueous buffer solution containing 30 mM trisodium citrate and 30 mM sodium tetraborate and adjusted the pH to a specific value with HCl. For buffers from a pH 9 to 11, we prepared an aqueous solution containing 30 mM CHES (*N*-cyclohexyl-2-aminoethanesulfonic acid) (Dojindo, Kumamoto, Japan) or 30 mM CAPS (*N*-cyclohexyl-3-aminopropanesulfonic acid) (Dojindo) and adjusted the pH with NaOH. When we mixed the buffer and a protein solution, the pH change was <0.1 units at 25°C. We used the Wolfram Mathematica 12.0 software (Wolfram Research, Champaign, IL, USA) to perform regularized maximum likelihood calculations to analyze the pH-dependent absorbance.

### Mammalian cell culture and transfection

We transfected mammalian cells with an expression vector using polyethylene imine (PEI Max 40K, #24765; Polysciences, Warrington, PA, USA) according to the manufacturer’s instructions. Just before microscopy observation, we rinsed the cells in the glass-bottom dish with 2 ml of Gibco Dulbecco’s Modified Eagle’s Medium (DMEM)/F12 containing *L*-glutamine and 2-[4-(2-hydroxyethyl)-1-piperazinyl]-ethanesulfonic acid (HEPES) but not phenol-red (11039021; Thermo Fisher Scientific) and subsequently added 2 ml of the same medium to the dish. For the SPoD-OnSPAN observation (see Time-lapse SR imaging of cells expressing Kohinoor2.0 in Results and discussion), we supplemented the medium with a final concentration of 10% (v/v) fetal bovine serum (S1780; Biowest, Riverside, MO, USA).

### Microscopy instruments

We observed the localization of Kohinoor2.0-fusion proteins in HeLa cells using an FV1000 confocal microscope with FluoView 4.0 software (Olympus, Hachioji, Japan) equipped with an oil-immersion microscope objective (magnification, ×60; numerical aperture (NA), 1.35) (UPlanSApo; Olympus). The wavelength of excitation and on-switching was 488 nm, while that of off-switching was 405 nm. The wavelength range of fluorescence detection was 500–600 nm.

We employed SPoD-OnSPAN [8,9] for SR imaging. A SPoD-OnSPAN microscope was built according to a previous study [9], composed of an inverted microscope (IX83; Olympus), an oil-immersion microscope objective (magnification, ×100; NA, 1.45) (UPLXAPO100XO; Olympus), a laser for excitation and on-switching (wavelength, 488 nm) (PC14584; Spectra Physics, Santa Clara, CA, USA), a laser for off-switching (wavelength, 405 nm) (OBIS 405LX-100-HS-WM; Coherent, Santa Clara, CA, USA) and a complementary metal-oxide-semiconductor (CMOS) camera (ORCA-Flash4.0 V2 C11440-22CU; Hamamatsu Photonics). The IX83 microscope was equipped with a Z-drift compensator (IX3-ZDC2; Olympus), an ultrasonic motorized stage (IX3-SSU; Olympus) and a stage-top incubator (Tokai Hit, Fujinomiya, Japan) with the supply of air containing 5% CO_2_. For the observation of Kohinoor2.0 and Kohinoor, we specifically used a bandpass filter of FF01-525/45-25 (Semorock, Rochester, NY, USA) and a dichroic mirror of FF518-Di01-25 × 36 (Semrock). For the image reconstruction calculation of super-resolved time-lapse image data, we coded a program in C++ that performed almost the same operations as the image reconstruction program provided in our previous study [8] to significantly reduce the computation time. The executable file was generated by Intel C/C++ compiler (ver. 19.0, Intel, Santa Clara, CA, USA).

### Comparison of SPoD-OnSPAN images of Kohinoor2.0 and Kohinoor

We constructed an expression vector containing a gene of mCherry connected to P2A and LifeAct-Kohinoor2.0 (mCherry-P2A-LifeAct-Kohinoor2.0) and an expression vector containing a gene of mCherry connected to P2A and LifeAct-Kohinoor (mCherry-P2A-LifeAct-Kohinoor). P2A is a self-cleaving polypeptide [18]. The expression of the mCherry-P2A-LifeAct-Kohinoor2.0 gene in COS7 cells was expected to yield almost equimolar amounts of mCherry-P2A and LifeAct-Kohinoor2.0, and similarly, that of mCherry-P2A-LifeAct-Kohinoor gene was expected to yield mCherry-P2A and LifeAct-Kohinoor. For the excitation of mCherry, we introduced a 561-nm laser (Sapphire 561 LP; Coherent) into the SPoD-OnSPAN microscope (see Microscopy instruments). We performed SPoD-OnSPAN observation of COS7 cells which were cultured for 24–36 h after transfection with the expression vectors. We observed mCherry fluorescence excited at 561 nm through a bandpass filter of FF01-647/57-25 (Semrock). We read the mCherry fluorescence intensity at several points in each cell to choose a cell so that the fluorescence intensities from cells chosen in this experiment exhibited almost consistent intensities. Subsequently, we performed the SPoD-OnSPAN image acquisition of the chosen cell excited at 488 nm with a bandpass filter of FF01-525/45-25 (Semrock). The image data were applied to the image reconstruction calculation (see Microscopy instruments).

## Results and discussion

### Development of Kohinoor2.0

We performed directed evolution in order to create a pRSFP with improved photoproperties, including a higher fluorescence intensity compared to Kohinoor [7]. We used Kohinoor as a starting material to perform random mutagenesis by error-prone PCR and StEP [19] to produce a cDNA library of Kohinoor mutants. We transformed *E. coli* cells with the genes from the cDNA library to express mutant proteins and monitored the fluorescence of each colony through a bandpass filter (transmission wavelength, 522–550 nm) to screen >50 000 colonies on agar plates. We paid attention not only to the fluorescence intensity but also to the photoswitching speed and chromophore maturation in the screening. Ultimately, we picked up a mutant of Kohinoor-M40V/L153V/S162L/R170L/L185M/Y188N/E214V, which we denoted as Kohinoor2.0 (for the primary sequence, see Supplementary Fig. S1). Kohinoor2.0 showed improvements in fluorescence intensity, off-switching speed and maturation speed (see Photophysical and photochemical properties of Kohinoor2.0).

### Photophysical and photochemical properties of Kohinoor2.0

**Fig. 1.**
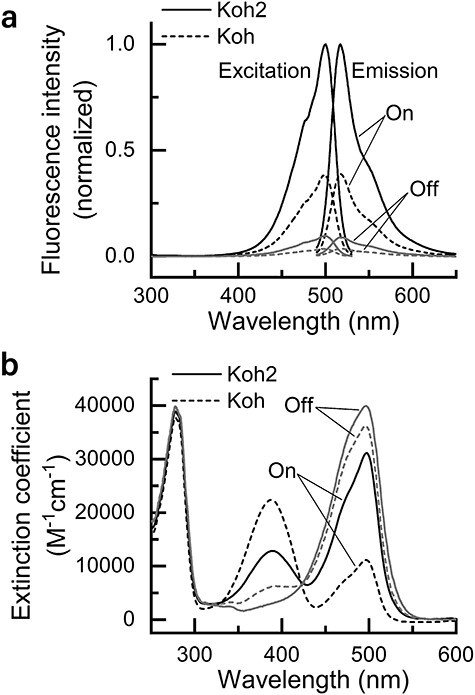
Fluorescence and absorption spectra of Kohinoor2.0 and Kohinoor at pH 7.4. (a) Fluorescence emission and excitation spectra of Kohinoor2.0 and Kohinoor in the on and off states at a concentration of 1 µM. Before measurement, the sample solutions were irradiated with an on-switching light at 475 ± 14 nm and 0.69 mW cm^−2^ or an off-switching light at 386 ± 12 nm at a power density of 0.69 mW cm^−2^ for 10 min. (b) Absorption spectra of Kohinoor2.0 and Kohinoor in the on and off states. The on- and off-switching was performed in the same conditions as in (a). The proteins were dissolved in a 20 mM 2-[4-(2-hydroxyethyl)-1-piperazinyl]-ethanesulfonic acid (HEPES)-NaOH buffer (pH 7.4). The temperature was 25°C. Koh2: Kohinoor2.0; Koh: Kohinoor.

Figure 1a shows the fluorescence spectra of purified Kohinoor2.0 and Kohinoor in the fluorescently bright and dark states, i.e. the on and off states. The excitation and emission peaks in the on state were observed at 500 and 516 nm, respectively, for both Kohinoor2.0 and Kohinoor. However, the peak emission intensity at 516 nm for Kohinoor2.0 was 2.6-fold higher than that for Kohinoor (Fig. 1a). This enhancement of fluorescence intensity in the on state was mainly attributed to a 2.8-fold higher extinction coefficient of Kohinoor2.0 (Fig. 1b), because the fluorescence quantum yields for both proteins were very close (Table 1). Additionally, the fluorescence excitation and emission peaks in the off state were also observed at 500 and 516 nm, respectively, for Kohinoor2.0 and Kohinoor (Fig. 1a). The ratio of the fluorescence intensity in the on state to that in the off state was measured to be 11.2 and 11.1 for Kohinoor2.0 and Kohinoor, respectively.

**Table 1.** Photophysical and photochemical parameters of Kohinoor2.0 and Kohinoor at neutral pH^a^

	Excitation/emission (nm)	Extinction coefficient^b^	Fluorescence quantum	Brightness	Maturation speed^c^		
	wavelengths (nm)	(M^−1^ cm^−1^)	yield		(*t*_1/2_, min)	Photoswitching rates^d^ (at 1 W cm^−2^) (s^−1^)	
						On-switching	Off-switching
Kohinoor2.0	500/516	31 200 ± 1300 (497 nm, on)	0.607 ± 0.002	19 300 ± 700	14.7 ± 0.9	7.4 ± 0.3 (*n *= 8)	29.5 ± 1.5 (*n *= 8)
		39 200 ± 500 (496 nm, off)					
Kohinoor	500/516	11 100 ± 300 (496 nm, on)	0.606 ± 0.002	6750 ± 200	36.8 ± 4.5	7.4 ± 0.1 (*n *= 6)	19.6 ± 0.8 (*n *= 6)
		36 200 ± 1500 (496 nm, off)					

a The data are shown in mean ± standard error (*n* = 3, unless specified).

b Determined from the absorbance at pH 7.4 and the chromophore concentration measured by the alkaline denaturation method [21].

c Explained in section S3 of Supplementary Materials.

d Explained in section S2 of Supplementary Materials. The values were measured in HeLa cells.

The absorption spectra of Kohinoor2.0 and Kohinoor in the on state showed that 390- and 500-nm absorption bands were present at a neutral pH (pH 7.4) (Fig. 1b). However, only the 500-nm absorption band contributed to the fluorescence emission (Fig. 1a), which is consistent with our previous report on Kohinoor [7]. The 390- and 500-nm absorption bands are most likely to be attributed to the neutral form of the phenolic group ((neutral) chromophore phenol) and the anionic form of the phenolic group ((anionic) chromophore phenolate) in the chromophore, respectively, in the cis configuration, according to the structural knowledge of Padron [22], from which Kohinoor was derived. Thus, the presence of both 390- and 500-nm absorption bands indicates that the neutral and anionic forms of the chromophore phenolic group are in equilibrium at neutral pH. In fact, if the two forms were not in equilibrium, then the absorbance of the 390- and 500-nm bands should not change in a pH-dependent manner (see pH-dependent conformation states and properties of Kohinoor2.0 and Kohinoor).

We also measured the speeds of photoswitching, chromophore maturation and photobleaching of Kohinoor2.0 and Kohinoor. The on-switching rate was almost the same between Kohinoor2.0 and Kohinoor fused with vimentin, an intermediate filament protein, in COS7 cells, but the off-switching rate was 1.5-fold higher for Kohinoor2.0 (Table 1; Supplementary Section S2). In SR observation by SPoD-OnSPAN (see Time-lapse SR imaging of cells expressing Kohinoor2.0) [8,9], although the relationship of spatial resolution with photoswitching rates and fluorescence intensity is not straightforward, a faster off-switching rate is expected to bring about a higher polarization angle-narrowing of the on state of pRSFP in analogy to SPoD-ExPAN [6]. The higher polarization angle-narrowing was shown to improve detectability of fine structures embedded in specimen and successful image reconstruction [6,8]. However, because faster off-switching could bring about lower fluorescence intensity in SR observation involving off-switching, the trade-off between them should also be considered. In addition, because a photoswitching rate is proportional to power density in the linear response regime of photoswitching, the faster photoswitching rate suggests that a lower power density of light at 405 nm should be enough to perform off-switching when using Kohinoor2.0, which should achieve less phototoxicity in live cells in SR imaging. The faster off-switching for Kohinoor2.0, irrespective of a lower extinction coefficient at 405 nm, was attributed to a higher off-switching quantum yield for Kohinoor2.0 than that for Kohinoor (Supplementary Table S1). The speed of chromophore maturation for Kohinoor2.0 was 2.5-fold faster than that for Kohinoor as measured through the time development of fluorescence intensity from immature Kohinoor2.0 and Kohinoor in an air-saturated condition (Supplementary Section S3). In the chromophore maturation process, the oxidation of cyclized chromophore precursor is thought be the rate-limiting step [23]. The faster chromophore maturation for Kohinoor2.0 would be propitious for assured fluorescent labeling with less defect. The photobleaching rate constant for Kohinoor2.0 fused with vimentin in COS7 cells with irradiation at 488 nm and 1 W cm^−2^ was 1.3-fold higher than that for Kohinoor (Supplementary Section S4). However, the photobleaching quantum yield of Kohinoor2.0 was 1.8-fold lower than that of Kohinoor (Supplementary Table S2), meaning that the probability of photobleaching per excitation/de-excitation cycle was lower for Kohinoor2.0 than Kohinoor.

**Fig. 2.**
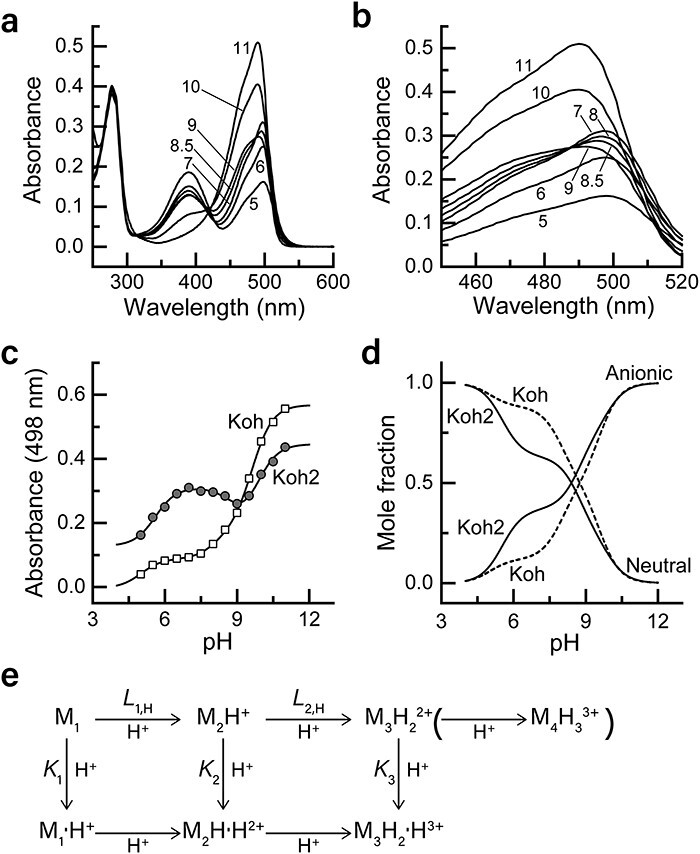
pH-dependent absorption of Kohinoor2.0 and Kohinoor in the on state and an equilibrium model which describes the pH dependence. (a) pH-dependent absorption spectra of Kohinoor2.0 in the on state at a concentration of 10 µM. Before measurement, we performed on-switching using the same procedure as in Fig. 1. The temperature was 25°C. (b) An expanded view of the pH dependence of the 500-nm band. (c) pH profiles of the absorption at 498 nm for Kohinoor2.0 and Kohinoor. The solid lines are derived from a model presented in (e). (d) pH-dependent mole fractions of the anionic chromophore phenolate and the neutral chromophore phenol computed from the model in (e) (Supplementary Section S6). (e) A model which describes the pH profiles of Kohinoor2.0 and Kohinoor. The vertical arrows indicate the protonation to the anionic chromophore phenolate. The horizontal arrows indicate protonation which occurs at non-chromophore phenolate sites. M*_i_*H*_i _*_−1_(*i* = 1, 2, 3) in the top row represents *i*-th conformation state of Kohinoor2.0 or Kohinoor with (*i *− 1) bound proton(s) at non-chromophore phenolate site(s) and an ion valence of +(*i *− 1) relative to the M_1_ state. M*_i_*H*_i _*_−1_•H in the bottom row represents the M*_i_*H*_i _*_−1_ state with a proton bound to the chromophore phenolate (‘•H’ in the scheme), where the relative ion valence becomes + *i*. Additionally, we suppose the M_4_H_3_^3+^ state to account for the significant presence of the 500-nm absorption band of Kohinoor2.0 even at pH 5.0 (see panel (a)), although this state was not included in the model function Eq. (4). The numbers in (a) and (b) refer to pH. ‘Anionic’ and ‘neutral’ in (d) denote the anionic chromophore phenolate and neutral chromophore phenol, respectively. Koh2: Kohinoor2.0; Koh: Kohinoor.

### pH-dependent conformation states and properties of Kohinoor2.0 and Kohinoor

We analyzed the pH-dependent absorption spectra of purified Kohinoor2.0 and Kohinoor to determine how the fluorescence intensity was improved. Since the data in our previous report implied intricate pH dependence of the fluorescence intensity in Kohinoor [7], we decomposed the observed absorbance into the individual conformation states. The absorption spectra of Kohinoor2.0 at 25°C between pH 5 and pH 11 showed that the absorbance of 500- and 390-nm absorption bands was negatively correlated (Fig. 2a). This indicates that the fraction of the anionic chromophore phenolate increases as the pH increases, whereas that of the neutral chromophore phenol decreases as the pH increases [4]. Furthermore, upon a closer look at the 500-nm absorption band, we noticed an intricate change in the spectral shape (Fig. 2b): the peak wavelengths were 498 and 491 nm at pH 5 and pH 11, respectively, and a shoulder was observable around 470 nm above pH 8. Additionally, an isosbestic point at 488 nm was observed among the spectra between pH 7 and pH 9. These data suggest that there exist at least three distinct conformation states in relation to the electronic states of the anionic chromophore phenolate as long as the 500-nm absorption band is concerned. Additionally, the pH profiles of 498-nm absorbance from Kohinoor2.0 and Kohinoor (Fig. 2c) could not fit a simple model of single protonation equilibrium, which underpins our hypothesis that both proteins are in multiple equilibria of at least three distinctive conformation states.

We devised a model to describe the conformation transitions and the protonation of the chromophore phenolate based upon the results above. We assumed that the three conformation states of Kohinoor2.0 and Kohinoor were involved in the change in the absorbance of the 500-nm band, with each conformation state bearing a unique value of binding constant for the protonation of the chromophore phenolate (Fig. 2e). Furthermore, we assumed that the protonation to the dissociative groups, except for the chromophore phenolate, was associated with the conformational change. Herein, let M_1_, M_2_H^+^ and M_3_H_2_^2+^ be the conformation states of Kohinoor2.0 or Kohinoor with the anionic chromophore phenolate, where H represents protons bound to non-chromophore phenolate sites and the superscripts represent the ion valence relative to the M_1_ state. Let M_1_•H^+^, M_2_H•H^2+^ and M_3_H_2_•H^3+^, respectively, be the corresponding conformation states with the neutral chromophore phenol, where ‘•H’ represents a proton bound to the chromophore phenolate and the superscripts denote their ion valence. The subscripts of M are for indexing the distinct conformation states in reference to binding constants, absorbance and extinction coefficients (see Eqs (1-4); Table 2). Note that M_1_, M_2_H^+^ and M_3_H_2_^2+^ are highly fluorescent and M_1_•H^+^, M_2_H•H^2+^ and M_3_H_2_•H^3+^ are fluorescently dark. Thereby, we express the equilibrium constants of these conformation changes as

(1) \begin{equation*}{L_{1,{\rm{H}}}} = {{[{{\rm{M}}_2}{{\rm{H}}^ + }]} \over {[{{\rm{M}}_1}]x}}\;{\rm and}\;{L_{2,{\rm{H}}}} = {{[{{\rm{M}}_3}{{\rm{H}}_2}^{2 + }]} \over {[{{\rm{M}}_2}{{\rm{H}}^ + }]x}}\end{equation*}

where *x* = [H^+^] and *L*_1,H_ > *L*_2,H_. Let *K*_1_, *K*_2_ and *K*_3_ be the protonation equilibrium constants of the chromophore phenolate for M_1_, M_2_H^+^ and M_3_H_2_^2+^, respectively. We express the equilibrium constants as

(2) \begin{equation*}{K_1} = {{[{{\rm{M}}_1} \cdot {{\rm{H}}^{\rm{ + }}}]} \over {[{{\rm{M}}_1}]x}},{K_2} = {{[{{\rm{M}}_2}{\rm{H}} \cdot {{\rm{H}}^{{\rm{2 + }}}}]} \over {[{{\rm{M}}_2}{{\rm{H}}^ + }]x}},{\rm and}\;{K_3} = {{[{{\rm{M}}_3}{{\rm{H}}_{\rm{2}}} \cdot {{\rm{H}}^{{\rm{3 + }}}}]} \over {[{{\rm{M}}_3}{{\rm{H}}_2}^{2 + }]x}}\end{equation*}

**Table 2.** Equilibrium parameters and extinction coefficients derived from the model in Fig. 2e (mean ± standard error, *n *= 3)

	log_10_*K*_1_	log_10_*K*_2_	log_10_*K*_3_	log_10_*L*_1,H_	log_10_*L*_2,H_	Extinction coefficient (M^−1^ cm^−1^)^a^					
						*ε*_1,A_	*ε*_2,A_	*ε*_3,A_	*ε*_1,N_	*ε*_2,N_	*ε*_3,N_
Kohinoor2.0	9.35 ± 0.09	8.62 ± 0.13	5.96 ± 0.07	9.80 ± 0.03	8.39 ± 0.08	41 100 ± 2000	28 900 ± 2100	87 100 ± 4400	$\sim$0	$\sim$0	13 300 ± 2600
Kohinoor	9.29 ± 0.03	8.24 ± 0.06	5.94 ± 0.03	9.27 ± 0.04	7.36 ± 0.05	55 300 ± 1500	34 300 ± 1800	81 200 ± 2700	$\sim$0	$\sim$0	$\sim$0

a The extinction coefficients are calculated by *ε* = *A*/*c*, where *A* is absorbance in Eq. (4) and *c* is a protein concentration.

where *K*_1_ > *K*_2_ > *K*_3_. Thus, in the present model, the M_1_ state is dominant at a high pH limit, and the M_3_H_2_•H^3+^ state is dominant at a low pH limit. Additionally, for simplicity of formulation, we let *P* be a binding polynomial [24], such that

(3) \begin{eqnarray*} P & \,\,\,\equiv\,\,\, & {{\sum\limits_i {[{\rm{M}}{{\rm{H}}_i}] + [{\rm{M}}{{\rm{H}}_i} \cdot {\rm{H}}]} } \over {[{{\rm{M}}_1}]}}\notag\\ &\!\!\!\,\,\,=& \!\!\!\!\!\,\,\, 1 \! + \! ({K_1} \! + {L_{{\rm{1,H}}}})x + ({K_2}{L_{{\rm{1,H}}}} + {L_{{\rm{1,H}}}}{L_{{\rm{2,H}}}}){x^2} + {K_3}{L_{{\rm{1,H}}}}{L_{{\rm{2,H}}}}{x^3}.\notag\\ \end{eqnarray*}

Hence, we have a model function which emulates the apparent absorbance *A*_model_ given by

(4) \begin{eqnarray*} {A_{{\rm{model}}}} \,\,\,\,\,\,&\!\!\!=\!\!\!&\,\,\,\,\,\, {1 \over {\sum {[{\rm{M}}{{\rm{H}}_i}] + [{\rm{M}}{{\rm{H}}_i} \cdot {\rm{H}}]} }}\Big[ {A_{1,{\rm{A}}}}[{{\rm{M}}_1}] + {A_{2,{\rm{A}}}}[{{\rm{M}}_2}{{\rm{H}}^ + }] \notag \\ && \,\,\,+ {A_{3,{\rm{A}}}}[{{\rm{M}}_3}{{\rm{H}}_2}^{2 + }] + {A_{1,{\rm{N}}}}[{{\rm{M}}_1} \cdot {{\rm{H}}^{\rm{ + }}}] + {A_{2,{\rm{N}}}}[{{\rm{M}}_2}{\rm{H}} \cdot {{\rm{H}}^{{\rm{2 + }}}}] \notag \\ && \,\,\,+ {A_{3,{\rm{N}}}}[{{\rm{M}}_3}{{\rm{H}}_{\rm{2}}} \cdot {{\rm{H}}^{{\rm{3 + }}}}] \Big]\notag\\ \,\,\,\,\,\,&\!\!\!=\!\!\!&\,\,\,\,\,\, {1 \over P}\left[ {A_{1,{\rm{A}}}} + {A_{2,{\rm{A}}}}{L_{{\rm{1,H}}}}x + {A_{3,{\rm{A}}}}{L_{{\rm{1,H}}}}{L_{{\rm{2,H}}}}{x^2} + {A_{1,{\rm{N}}}}{K_1}x\right. \notag\\ && \left. \,\,\,+ {A_{2,{\rm{N}}}}{K_2}{L_{{\rm{1,H}}}}{x^2} + {A_{3,{\rm{N}}}}{K_3}{L_{{\rm{1,H}}}}{L_{{\rm{2,H}}}}{x^3} \right] \end{eqnarray*}

where *A*_1,A_, *A*_2,A_, *A*_3,A_, *A*_1,N_, *A*_2,N_ and *A*_3,N_ are absorbance at a 100% mole fraction for M_1_, M_2_H^+^, M_3_H_2_^2+^, M_1_•H^+^, M_2_H•H^2+^ and M_3_H_2_•H^3+^, respectively. As explained in Supplementary Section S5, we performed regularized maximum likelihood calculations to fit *A*_model_ to the measured data to search for optimal values of the equilibrium constants and extinction coefficients (Fig. 2c, Table 2).

The 2.6-fold higher fluorescence intensity of Kohinoor2.0 compared to Kohinoor at a neutral pH (Fig. 1a) is likely to be ascribed to the stabilization of the anionic chromophore phenolate. In analogy to Padron [22], the chromophore phenolic group of Kohinoor2.0 and Kohinoor which can fluoresce would be in the form of the anionic phenolate. Using the parameter values derived from the present analysis (Table 2), we computed the mole fractions of the anionic chromophore phenolate and the neutral chromophore phenol as a function of pH (Fig. 2d; Supplementary Section S6 and Fig. S5). The data show that the mole fraction of the anionic chromophore phenolate in Kohinoor2.0 is 1.9-fold higher than that for Kohinoor at pH 7.4, indicating a 1.9-fold higher stability of the anionic chromophore phenolate in Kohinoor2.0 than in Kohinoor. According to Table 2 and Supplementary Fig. S5, the M_3_H_2_^2+^ state is the primary contributor to fluorescence intensity at neutral pH, and its extinction coefficient *ε*_3_ is very close between Kohinoor2.0 and Kohinoor. The logarithm of the protonation binding constant for the transition from the M_2_H^+^ state to the M_3_H_2_^2+^ state, log_10_*L*_2,H_, was higher by 1.0 units for Kohinoor2.0 than that for Kohinoor, leading to higher stabilization of the M_3_H_2_^2+^ state in Kohinoor2.0 (Supplementary Fig. S5a). Thus, the data suggest that the mutations introduced into Kohinoor2.0 bring about the higher stabilization of the fluorescent M_3_H_2_^2+^ state at neutral pH, which leads to the higher extinction coefficient of the 500-nm absorption band and, thereby, a higher fluorescence intensity.

Although we have found the multiple equilibrium behavior of the chromophore phenolate in Kohinoor2.0 and Kohinoor, the relationship of the pH-dependent absorption spectrum to the configurations of the amino acids inside the β-barrel remains elusive. Previously, Brakemann *et al.* [25] reported that Padron 0.9, which was derived from Padron like Kohinoor2.0 and Kohinoor, showed a complicated pH titration behavior, although they did not analyze the pH-dependent equilibrium in detail. Brakemann *et al.* also performed quantum mechanics/molecular mechanics (QM/MM) calculations to determine the free energy change between the neutral and anionic forms of chromophore phenolate [25]. They suggested that the p*K*_a_ of the chromophore phenolate could shift by  $\sim$3 units, depending on the protonation states of the surrounding dissociative groups (His-193, Glu-144 and Glu-211, which are also conserved in Kohinoor2.0 and Kohinoor) and the configuration of the hydrogen bonding network around the chromophore [25]. If we take the result of their QM/MM study [25] into consideration, it suggests that the protonation of His-193 and/or Glu-144 or Glu-211 may be involved in *L*_1,H_ and *L*_2,H_, such that the binding constant of the chromophore phenolate splits into several values in a pH-dependent manner. To our knowledge, this is the first report proposing an explicit model which describes the multiple equilibria in the protonation of the chromophore phenolate in green fluorescent protein-like fluorescent proteins. Furthermore, we suggest that a detailed re-consideration of the pH-dependent behavior could be a vital clue for the improvement of fluorescent proteins in the future.

We also examined pH dependence of fluorescence emission and photoswitching rates. Kohinoor2.0 excited at 488 nm showed higher fluorescence intensity than Kohinoor below pH 9, but showed lower fluorescence intensity above pH 9 (Supplementary Section S7 and Fig. S6), being similar with the pH profile of absorbance (Fig. 2c). However, the detailed pH behavior of fluorescence was slightly different from that of the 500-nm band absorbance, suggesting that the pH behavior of the fluorescence may reflect not only the populations of the conformation states with the ground state chromophore but also the relaxation of the chromophore and its environment during the excited state [26] (Supplementary Section S7). The rates of on-switching and off-switching of purified Kohinoor2.0 and Kohinoor were also found to be dependent on pH (Supplementary Section S8). The on-switching rates increased with pH (Supplementary Fig. S7d). The off-switching rates were observed to correlate with the pH-dependent populations of the conformation states, M_1_•H^+^, M_2_H•H^2+^ and M_3_H_2_•H^3+^ (Supplementary Fig. S7e and f).

### Comparison of fluorescence intensity between Kohinoor2.0 and Kohinoor in the imaging of HeLa cells

**Fig. 3.**
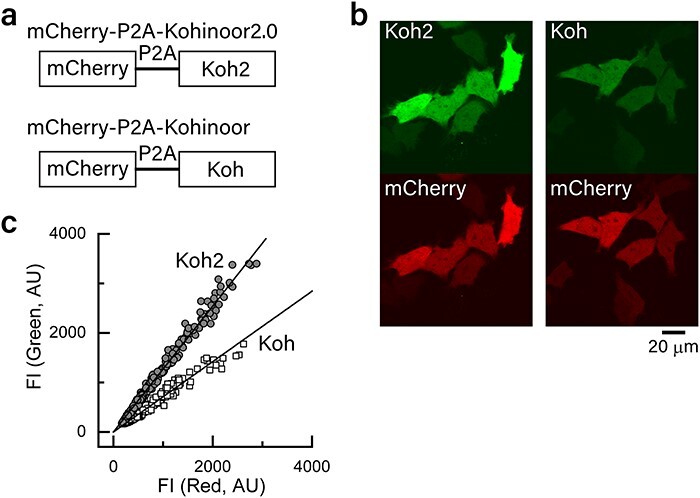
Comparison of fluorescence intensity between Kohinoor2.0 and Kohinoor in HeLa cells. (a) Schematic diagram of genes of mCherry-P2A-Kohinoor2.0 and mCherry-P2A-Kohinoor, which were expressed in HeLa cells. (b) Images of HeLa cells expressing the genes shown in (a) as observed using an FV1000 confocal microscope. For both cell samples, green fluorescence (Kohinoor2.0 or Kohinoor) was observed at an excitation of 488 nm and 10% of maximum laser power and red fluorescence (mCherry) was observed at an excitation at 543 nm and 10% of maximum laser power. (c) Plots of the green fluorescence intensity against red fluorescence intensity from each HeLa cell. The solid lines show linear regression to derive the green fluorescence intensity relative to the red fluorescence intensity. The cells were incubated for 16 h before observation. The observation was performed at room temperature. arbitrary unit; FI: fluorescence intensity; Koh2: Kohinoor2.0; Koh: Kohinoor.

We compared the fluorescence intensity of Kohinoor2.0 and Kohinoor in HeLa cells. We used the expression vectors encoding mCherry-P2A-Kohinoor2.0 and mCherry-P2A-Kohinoor, in which Kohinoor2.0 and Kohinoor are fused to a red fluorescent protein of mCherry [17] via a self-cleaving polypeptide of P2A [18] (Fig. 3a). We expected that HeLa cells transfected with the vectors would produce equimolar amounts of Kohinoor2.0 or Kohinoor and mCherry-P2A and the C-terminal of mCherry-P2A would be disconnected from Kohinoor2.0 or Kohinoor. Thus, Kohinoor2.0 or Kohinoor and mCherry-P2A were expected to be separate in the cells. In fact, using a confocal microscope, we were able to find transfected HeLa cells that exhibited green fluorescence from Kohinoor2.0 or Kohinoor (upper micrographs in Fig. 3b), as well as red fluorescence from mCherry (lower micrographs in Fig. 3b). In the images of the HeLa cells, we measured the intensity of green fluorescence from Kohinoor2.0 or Kohinoor and that of red fluorescence from mCherry in each cell. The green fluorescence intensity was plotted against the red fluorescence intensity (Fig. 3c). The data show that although the fluorescence intensity of the fluorescent proteins varies considerably between cells, the fluorescence intensity from Kohinoor2.0 and Kohinoor (the vertical axis, Fig. 3c) is linearly correlated with that of mCherry (horizontal axis, Fig. 3c). This suggests that even though the expression levels of the fluorescent proteins were different from cell to cell, the molar ratio of Kohinoor2.0 or Kohinoor to mCherry was almost constant over the cells, presumably near to unity, according to the gene design (Fig. 3a). Thus, by taking the fluorescence intensity of mCherry as a measure of the expression levels for Kohinoor2.0 and Kohinoor, we compared the linear regression of the plots in Fig. 3c. The result showed that the slope of the plot for Kohinoor2.0 by linear regression was 1.8-fold larger than that for Kohinoor, suggesting a 1.8-fold higher fluorescence intensity in Kohinoor2.0 compared to Kohinoor in HeLa cells, which was fairly similar to the results of the fluorescence intensity of the purified proteins (Table 1). Although the relative fluorescence intensity of Kohinoor2.0 may be different between the data from HeLa cells and purified proteins to a small extent, this may be due to differences in the environment of the fluorescent proteins: cytoplasm in HeLa cells and aqueous buffer solution.

### Time-lapse SR imaging of cells expressing Kohinoor2.0

**Fig. 4.**
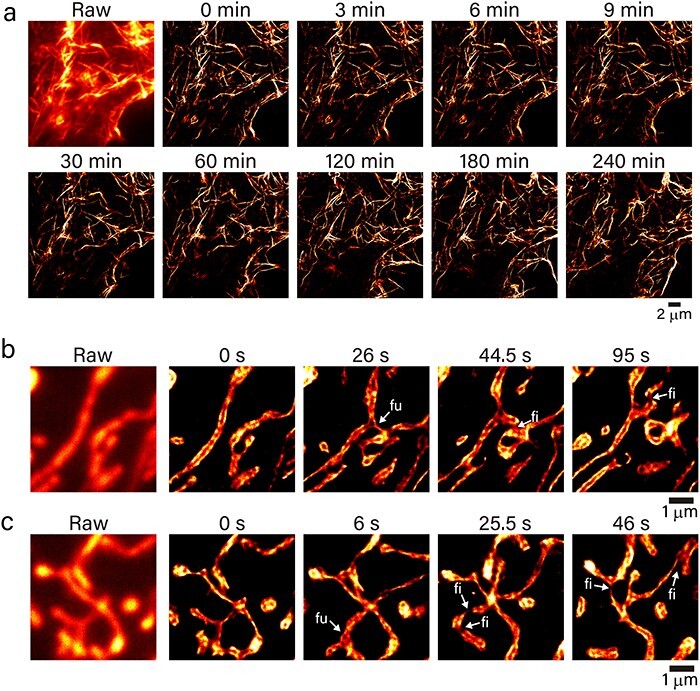
Time-lapse super-resolution observation of Kohinoor2.0 in live COS7 cells by super-resolution polarization demodulation/on-state polarization angle narrowing (SPoD-OnSPAN). (a) Extract from the time-lapse observation of actin filaments labeled with LifeAct-Kohinoor2.0. An image dataset composed of 18 raw image frames to reconstruct a super-resolved image was taken at an exposure time of 0.3 s for each raw frame within 6 s. The image dataset was acquired every 3 min. The irradiation power density was 0.99 and 0.67 W cm^−2^ at 488 and 405 nm, respectively. (b, c) Extract from the time-lapse observation of mitochondria labeled with tsCOX8-Kohinoor2.0. Each image dataset composed of 9 raw image frames to reconstruct a super-resolved image was taken at an exposure time of 0.047 s for each raw frame within 0.5 s. The irradiation power density was 1.2 and 0.81 W cm^−2^ at 488 and 405 nm, respectively. The observation was performed on an in-house built SPoD-OnSPAN microscope at 37°C in air containing 5% CO_2_.

Taking advantage of the improvement to Kohinoor2.0, we performed a time-lapse SR observation with Kohinoor2.0 by SPoD-OnSPAN [8,9]. We confirmed that Kohinoor2.0-fusion proteins properly showed localization in HeLa cells by confocal microscopy (Supplementary Section S9), and we tried SPoD-OnSPAN observation of actin filaments and mitochondria in COS7 cells. Figure 4a shows a time series of intermittently observed SR images of actin filaments labeled with LifeAct-Kohinoor2.0 in a COS7 cell taken by SPoD-OnSPAN. It should be noted that we performed the long-term SPoD-OnSPAN observation at illumination power density of as low as 0.99 and 0.67 W cm^−2^ at 488 and 405 nm, respectively. The cell was observed to last and maintain the actin filament network without an indication of phototoxicity for as long as 4 h. Because we took 6 s to take 18 raw image frames for each super-resolved image and repeated this data acquisition every 3 min for 4 h, the acquisition time amounted to 480 s. The videos of the raw and SPoD-OnSPAN images are provided in the Supplementary Materials (Supplementary videos, Fig.4a-RawAvg-LifeAct.tif and Fig.4a-SPoD-OnSPAN-LifeAct.tif; Supplementary Section S11). A typical spatial resolution in the SPoD-OnSPAN observation of Kohinoor2.0 was 50–70 nm as measured from the full widths at half maximum of actin filaments labeled with LifeAct-Kohinoor2.0 (Supplementary S10, Fig. S9e). Although this spatial resolution may be comparable to or lower than those of stimulated emission depletion (STED), RESOLFT and single-molecule localization microscopy (SMLM), the illumination power density used in the present SPoD-OnSPAN observation was several orders of magnitude lower than that used in these SR techniques [27]. The much lower illumination power density in the SPoD-OnSPAN observation should have led to low phototoxicity and photobleaching. In addition, although SIM could achieve SR imaging at a low illumination power density comparable to that of SPoD-OnSPAN, the improvement in spatial resolution is limited up to a factor of 2 compared to the diffraction limit of light [28]. Accordingly, the present SPoD-OnSPAN provides SR imaging with much lower phototoxicity than STED, RESOLFT and SMLM and a higher spatial resolution than SIM.

**Fig. 5.**
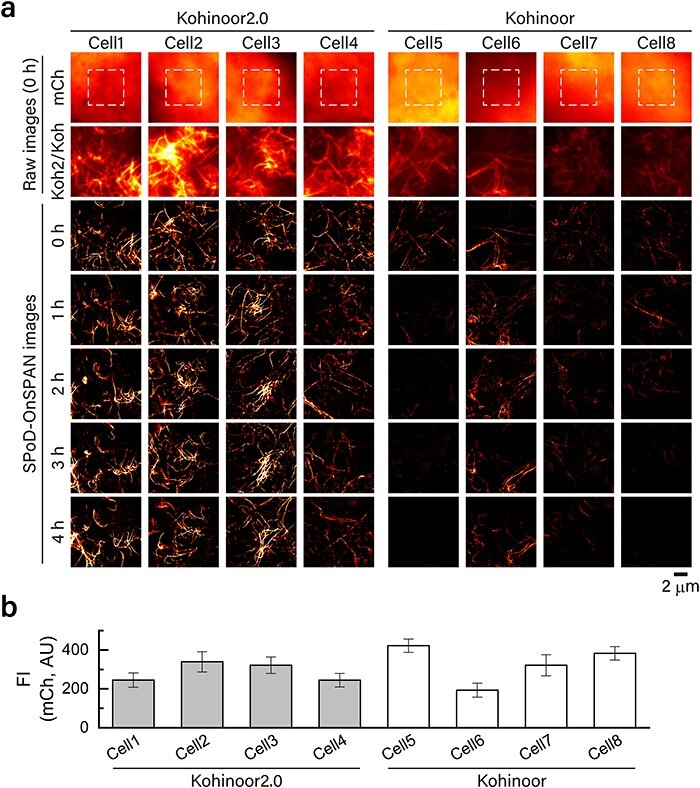
Comparison of Kohinoor2.0 and Kohinoor in super-resolution polarization demodulation/on-state polarization angle narrowing (SPoD-OnSPAN) observation at similar expression levels in COS7 cells. (a) Fluorescence images of cells expressing mCherry-P2A-LifeAct-Kohinoor2.0 (cells 1–4) and mCherry-P2A-LifeAct-Kohinoor (cells 5–8). The images in the row of mCh are ones of mCherry-P2A through a bandpass filter of FF01-647/57-25 (Semrock). The images in the row of Koh2/Koh are ones of LifeAct-Kohinoor2.0 and LifeAct-Kohinoor through a bandpass filter of FF01-525/45-25 (Semrock). The images in the rows of ‘SPoD-OnSPAN images’ are reconstructed super-resolved images taken from the time-lapse observation by SPoD-OnSPAN. Images in each column were taken from the same position and the same focal depth in the cell. (b) Fluorescence intensity of mCherry in cells 1–8. The average fluorescence intensity was taken from regions (enclosed by the dashed lines) of the images in the row mCh in panel (a). The error bars represent the standard deviation of pixel-wise intensity in the regions.

We also observed mitochondria labeled with tsCOX8-Kohinoor2.0 in COS7 cells using SPoD-OnSPAN. Since the mitochondria were found to be highly mobile (magnification, ×330 at the faceplate of the camera), we used SPoD-OnSPAN at a higher time-resolution to obtain super-resolved movies with a frame rate of 2 Hz. When we examined the reconstructed time-lapse super-resolved images, we noticed that the mitochondria underwent fusion and fission at times. Figure 4b and c shows typical examples of fusion, in which two mitochondria are combined (labeled in ‘fu’), and fission, in which a mitochondrion is split into two (labeled in ‘fi’), despite these processes not being clear in the raw images (see Supplementary videos, Fig.4c-RawAvg-Mt.tif and Fig.4c-SPoD-OnSPAN-Mt.tif; Supplementary Section S11). Although mitochondrial fusion and fission are well-known, the details of their molecular mechanisms remain elusive [29,30]. Additionally, in the present SPoD-OnSPAN observation, the borders of mitochondria were occasionally observed to show bright fluorescence signal (in particular, Fig. 4b). The tag of tsCOX8 polypeptide is known to direct its transport into mitochondrial matrix [31,32], but the localization of tsCOX8-fusion protein to mitochondrial matrix was reported to be affected by folding latency of fused proteins [33], suggesting that the transport of a rigidly folded protein fused with tsCOX8 to mitochondrial matrix across the inner membrane could be retarded. Thus, the bright fluorescence signal around the borders of mitochondria may reflect tsCOX8-Kohinoor2.0 attached on the inner membrane and waiting for transport. In addition, the polarization-induced modulation of fluorescence excitation, on-switching and off-switching in the present SPoD-OnSPAN observation was performed at a period of 0.25 s with on- and off-switching time constants of $\sim$10^−1^ s (Table 1). Thus, according to the principle of SPoD-OnSPAN, a fluorescent object composed of highly immobilized Kohinoor2.0 molecules exhibiting a rotational correlation time of >0.25 s could lead to high contrast in the reconstructed image, whereas freely rotating Kohinoor2.0 molecules should lead to low contrast. This may also underpin our speculation that the bright fluorescence around the border of the mitochondria reflects tsCOX8-Kohinoor2.0 attached on the inner membrane. Until now, the SR imaging of mitochondria dynamics has been performed by RESOLFT, STED, stochastic optical reconstruction microscopy and SIM [34–39]. It should be noted that the present SR imaging by SPoD-OnSPAN and Kohinoor2.0 was able to be performed at a very low-power density of irradiation lights ($\sim$1 W cm^−2^) alongside fairly high spatial and time resolutions. In contrast, most of the other SR imaging techniques require a power density that is several orders of magnitude higher, although mitochondria are known to be susceptible to phototoxicity [39]. In the future, with the combination of Kohinoor2.0, SPoD-OnSPAN and state-of-the-art image reconstruction techniques (e.g. deep neural networks [40]), we should be able to achieve real-time SR imaging of various cellular processes, including mitochondria dynamics. In particular, if the SR imaging with Kohinoor2.0 is applied to experiments, such as the specific labeling of key proteins, multi-color imaging and protein tracking with a photocontrollable fluorescent protein, we may be able to gain better insights into the molecular mechanisms of mitochondria dynamics.

### Comparison of SPoD-OnSPAN images of Kohinoor2.0 and Kohinoor at similar expression levels

We compared SPoD-OnSPAN observation of Kohinoor2.0 and Kohinoor in COS7 cells at similar expression levels. We prepared COS7 cells transfected with an expression vector containing a gene of mCherry-P2A-LifeAct-Kohinoor2.0 or mCherry-P2A-LifeAct-Kohinoor. Similar to cells transfected with mCherry-P2A-Kohinoor2.0 or mCherry-P2A-Kohinoor (Fig. 3), we expected that the transfected cells yielded almost equimolar amounts of mCherry-P2A and LifeAct-Kohinoor2.0 or LifeAct-Kohinoor. Thus, we took the mCherry fluorescence as a measure of the expression levels of Kohinoor2.0 and Kohinoor. By epi-fluorescence microscopy observation with excitation at 561 nm, we confirmed that mCherry nonspecifically distributed in COS7 cells (the row of mCh in Fig. 5a), indicating that mCherry-P2A was mostly free from actin filaments. In this experiment, as shown in Fig. 5b, we chose cells that showed average mCherry fluorescence intensities around 250 AU (square regions in the images in the row of mCh in Fig. 5a). In contrast with the nonspecific distribution of mCherry, Kohinoor2.0 and Kohinoor excited at 488 nm were observed to localize along actin filaments, indicating that LifeAct-Kohinoor2.0 and LifeAct-Kohinoor bound to the actin filaments (the row of Koh2/Koh in Fig. 5a). In addition, according to this data, we also confirmed that the fluorescence intensities of Kohinoor2.0 were higher than those of Kohinoor (the row of Koh2/Koh in Fig. 5a), notwithstanding the similar mCherry fluorescence intensities (Fig. 5b), and presumably, notwithstanding similar expression levels of Kohinoor2.0 and Kohinoor. Note that, for comparison, the images in each row of mCh or Koh2/Koh (Fig. 5a) are shown at the same settings of brightness and contrast. Furthermore, we performed time-lapse SPoD-OnSPAN observation of the same cells. We took 6 s to acquire 18 raw image frames that were used to reconstruct a super-resolved image, and repeated this acquisition sequence every 3 min for 4 h, leading to totally 480-s-long observation. As shown in the rows of ‘SPoD-OnSPAN images’ (Fig. 5a), LifeAct-Kohinoor2.0 typically continued to show a clearer image of the actin filaments with higher fluorescence intensity for a longer time than LifeAct-Kohinoor. This would be basically because of the higher fluorescence intensity of Kohinoor2.0. Note that for comparison, we observed the cells in the same observation condition, and the images in the rows of ‘SPoD-OnSPAN images’ (Fig. 5a) are also shown at the same settings of brightness and contrast. Thus, the data demonstrate the advantage of Kohinoor2.0 over Kohinoor in SR imaging.

## Conclusions

In this study, we developed a new pRSFP Kohinoor2.0 from Kohinoor using directed protein evolution. Kohinoor2.0 shows a 2.6-fold higher fluorescence intensity, 1.5-fold faster off-switching and 2.5-fold faster chromophore maturation than Kohinoor. These improved properties of Kohinoor2.0 should be advantageous to time-lapse SR imaging with a low phototoxicity in live cells, a high time-resolution and a better image quality. The higher fluorescence intensity of Kohinoor2.0 at a neutral pH was mainly attributed to the higher extinction coefficient of the 500-nm absorption band. The analysis of the pH dependence of the 500-nm absorption band revealed that Kohinoor2.0 was in multiple equilibria with three distinct conformation states, each of which showed a unique binding constant for the protonation to the anionic chromophore phenolate. It is suggested that the mutations introduced to Kohinoor2.0 modified the interplay of the amino acids surrounding the chromophore, such that the anionic chromophore phenolate, which exhibits the 500-nm absorption band in Kohinoor2.0, is more stabilized than that in Kohinoor. Using Kohinoor2.0 with our SR imaging technique of SPoD-OnSPAN, we demonstrated the SR imaging of the actin filament network in mammalian cells for as long as 4 h with a total acquisition time of 480 s without any indication of phototoxicity. Furthermore, we demonstrated the SR imaging of the mitochondria dynamics at a time resolution of 0.5 s, in which the fusion and fission processes were clearly visualized. Accordingly, in the present study, we demonstrated that SPoD-OnSPAN observation with Kohinoor2.0 allows for the SR imaging of cellular dynamics with a very low dose of light. In the future, Kohinoor2.0 could be used as an indicator of physiological functions, such as Ca^2+^ indicators for SR imaging. This is likely to pave the way for the visualization of the details of not only fine structures, but also of the physiological functions of cells.

## Supplementary Material

dfab001_Supp
