# Supplementary material for: A photoswitchable fluorescent protein for hours-time-lapse and sub-second-resolved super-resolution imaging
Source: Microscopy (Oxf). 2021 Jan 22;70(4):340–52. doi: 10.1093/jmicro/dfab001 (PMC8350982; doi:10.1093/jmicro/dfab001)
Supplement: dfab001_Supp [file dfab001_supp.zip › MICRO-2020-00085-MS-Supplementary.docx]

**Supplementary materials**

A photoswitchable fluorescent protein for hours-time-lapse and sub-second-resolved super-resolution imaging

Tetsuichi Wazawa, Ryohei Noma, Shusaku Uto, Kazunori Sugiura,

Takashi Washio, and Takeharu Nagai

**S1. The primary structure of Kohinoor2.0**

Kohinoor2.0 was derived from Kohinoor with mutations of M40V/L153V/S162L/R170L/L185M/Y188N/E214V. The primary structures of Kohinoor2.0 and Kohinoor are shown in Fig. S1.

| **Kohinoor**  **Kohinoor2.0**  **Kohinoor**  **Kohinoor2.0**  **Kohinoor**  **Kohinoor2.0**  **Kohinoor**  **Kohinoor2.0**  **Kohinoor**  **Kohinoor2.0**  **Kohinoor**  **Kohinoor2.0** | **1**  **MSVIKPDMKI KLRMEGAVNG HPFAIEGVGL GKPFEGKQSM**  **MSVIKPDMKI KLRMEGAVNG HPFAIEGVGL GKPFEGKQSV**  **41**  **DLKVKEGGPL PFAYDILTMA FCYGNRVFAK YPENIVDYFK**  **DLKVKEGGPL PFAYDILTMA FCYGNRVFAK YPENIVDYFK**  **81**  **QSFPEGYSWE RSMIYEDGGI CIATNDITLD GDCYIYEIRF**  **QSFPEGYSWE RSMIYEDGGI CIATNDITLD GDCYIYEIRF**  **121**  **DGVNFPANGP VMQKRTVKWE PSTEKLYVRD GVLKSDGNYA**  **DGVNFPANGP VMQKRTVKWE PSTEKLYVRD GVVKSDGNYA**  **161**  **LSLEGGGHYR CDSKTTYKAK KVVQLPDYHD VVHHIEIKSH**  **LLLEGGGHYL CDSKTTYKAK KVVQMPDNHD VVHHIEIKSH**  **201**  **DRDYSNVNLH EHAEAHSGLP RQAK**  **DRDYSNVNLH EHAVAHSGLP RQAK** |
| --- | --- |

**Fig. S1.** Primary sequences of Kohinoor2.0 and Kohinoor. Mutations introduced into Kohinoor2.0 are underlined.

**S2. Speed of photoswitching in mammalian cells**

We examined the photoswitching rates of Kohinoor2.0 and Kohinoor in cells, according to our method [1]. To measure the photoswitching rates, we observed HeLa cells expressing Kohinoor2.0 or Kohinoor fused to vimentin, an intermediate filament protein, at 37°C on a fluorescence microscope (Fig. S2a). We irradiated the cells with a continuous light at 488 nm (1.06 W/cm2) for excitation and on-switching and a light at 405 nm (1.01 W/cm2) for off-switching in a cyclic sequence of 2-s irradiation followed by a 2-s pause (Fig. S2b). Thereby, when the irradiation at 405 nm was turned on, the fluorescence intensity from Kohinoor2.0 or Kohinoor decreased, indicating that the off-switching and the on-switching simultaneously took place. When the irradiation at 405 nm was turned off, the fluorescence intensity increased, indicating that the on-switching took place (Fig. S2b). The time trajectories of the fluorescence intensity in this observation were analyzed by pseudo-first order kinetics, as previously reported [1]. Accordingly, we determined the rates of on-switching and off-switching at power densities of 1 W/cm2 (Fig. S2c, Table 1). Although the on-switching rate was almost the same between Kohinoor2.0 and Kohinoor, the off-switching rate was 1.5-fold faster for Kohinoor2.0 than that for Kohinoor. This indicates that Kohinoor2.0 requires a lower power density for off-switching light at 405 nm than Kohinoor, and thereby, the lower power density at 405 nm for the observation of Kohinoor2.0 should give rise to less phototoxicity in cells. Thus, Kohinoor2.0 is likely to be more useful than Kohinoor in microscopy observation involving photoswitching processes.

We previously theorized the time change of the fluorescence intensity of a pRSFP with the on-switching/excitation light and the off-switching light [1]. We implicitly suggested that the fluorescence intensity of a pRSFP simultaneously irradiated with an on-switching/excitation light and an off-switching light asymptotically approaches to a value, *F*ON+OFF, given by

, (S1)

where *F*ON and *F*OFF are fluorescence intensities purely in the on and off states, respectively, and *k*ON and *k*OFF are rate constants of the on- and off-switching, respectively, at given power densities of the lights. Note that *k*ON and *k*OFF are approximated to be proportional to power densities of the switching lights if the power densities are low enough. By using Eq. (S1), *k*ON and *k*OFF values from Table 1, and the on/off contrasts (Fig. 1), a ratio *F*ON/*F*ON+OFF was calculated to be 3.6 and 2.9 for Kohinoor2.0 and Kohinoor. Alternatively, the ratio *F*ON/*F*ON+OFF was also calculated directly from the time trajectories of fluorescence intensity (Fig. S2b), which was 3.3 ± 0.3 (mean ± SD; *n* = 12) and 3.1 ± 0.4 (mean ± SD; *n* = 6) for Kohinoor2.0 and Kohinoor, respectively.

Furthermore, we determined the absorption cross-section and the quantum yields of on- and off-switching. The molecular absorption cross-section *σ* (cm2) is a hypothetical cross-sectional area of a molecule such that all the light interacting with the area is absorbed [2]. We equate a fraction of absorbed light intensity by the molecular absorption cross-section with that by Beer-Lambert law as follows

, (S2)

where *ε* (mol−1L cm−1) is an extinction coefficient, *N*A (mol−1) is the Avogadro constant, *c*FP (mol L−1) is a concentration of a fluorescent protein, *S* (cm2) is a cross-sectional area of a light beam incident on a protein solution, and *l* (cm) is a light path length in the protein solution [2,3]. Hence, taking the zeroth- and first-order terms from the expansion of the exponential in the second right hand, we have

. (S3)

Furthermore, the photoswitching quantum yield *φ*S was calculated from

, (S4)

where *h* (J s) is the Planck constant, *c* (m s−1) is the speed of light, and *k* (s−1) is a rate constant of photoswitching measured with a photoswitching light at a wavelength *λ* (nm) and a power density *P* (W cm−2) [3,4]. We calculated the absorption cross-section and photoswitching quantum yield from the parameter values that were taken from the data in Figure 1b and Table 1, and the results are shown in Table S1. The quantum yield of off-switching for Kohinoor2.0 was 2.3-fold higher than that for Kohinoor.


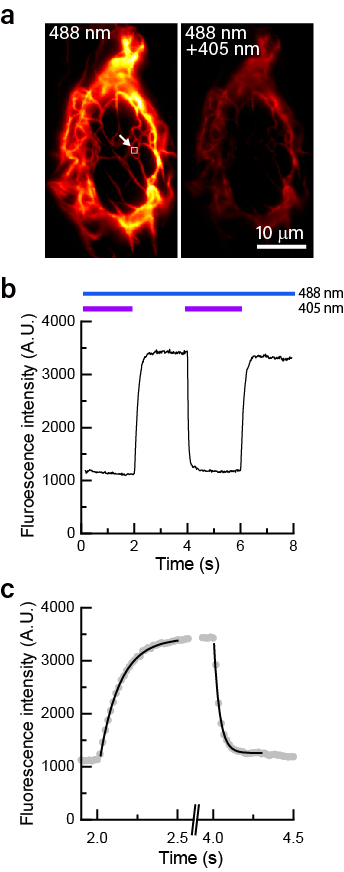


**Fig. S2.** Measurement of photoswitching rates of Kohinoor2.0 in a HeLa cell. (a) An image of vimentin intermediate filaments in a HeLa cell. (b) A time course of fluorescence intensity with cyclic irradiation at 405 nm and continuous irradiation at 488 nm. The fluorescence intensity was taken from the region indicated by an arrow in (c). The colored bars in the panel indicate the timings of the irradiation. (c) The time course of the panel (b) in an expanded scale. The solid curves are single exponential functions fit to the data points. The irradiation power density was 1.06 and 1.01 W/cm2 for 488 and 405 nm, respectively. The light at 405 nm was irradiated for an interval of 2 s every 4 s. The operation of the 405 nm laser was digitally modulated with a signal from a digital delay pulse generator (Sapphire Plus 9214+; Quantum Composers, Bozeman, MT, USA). The temperature was 37°C.

**Table S1.** Absorption cross-sections, and photoswitching quantum yield

|  | Absorption cross section  (×1017 cm2)* | |  | Photoswitching† | |
| --- | --- | --- | --- | --- | --- |
|  | Off state  (488 nm) | On state  (405 nm) |  | On-switching quantum yield  (488 nm) | Off-switching quantum yield  (405 nm) |
| Kohinoor2.0 | 14.4 ± 0.2 | 4.37 ± 0.29 |  | 0.0210  ± 0.0010 | 0.331  ± 0.027 |
| Kohinoor | 12.9 ± 0.5 | 6.57 ± 0.20 |  | 0.0236  ± 0.0010 | 0.147  ± 0.007 |

*mean ± SE; *n* = 3.

†The photoswitching rate constants in Table 1 were used to calculate the quantum yields. The standard errors were calculated by error propagation [5].

**S3. Speed of chromophore maturation**

We measured the chromophore maturation in Kohinoor2.0 and Kohinoor. The chromophore formation of Kohinoor2.0 and Kohinoor involving the three amino acids (Cys-62, Tyr-63, and Gly-64 [6]) consists of cyclization, dehydration, and oxidation [7]. The oxidation of the cyclized chromophore precursor was known to be the rate limiting step [8]. Thus, we measured the chromophore maturation by monitoring the time development of fluorescence intensity of immature Kohinoor2.0 and Kohinoor in an air-saturated condition using a previously reported method [9] with modifications. We subcloned a gene of Kohinoor2.0 or Kohinoor into a pBAD vector between BglII and HindIII sites. We transfected *E. coli* JM109 (DE3) cells with the vector to grow in a 10 mL LB medium with 0.1 mg/mL carbenicillin at 37°C until the absorbance at 595 nm of the culture reached 0.5. We induced protein expression by adding arabinose to a final concentration of 0.2% (w/v), and grew the culture for >4 h at 37°C under anaerobic condition in an AnaeroPack (Mitsubishi Gas Chemical, Tokyo, Japan). We subsequently collected the cells by centrifugation at 5,800 × *g* and 4°C for 5 min, and extracted the protein by ultrasonication on ice. The homogenized cells were centrifuged at 20,400 × *g* and 4°C for 5 min to collect the supernatant through a Miracloth filter (Millipore, Burlington, MA). We then immediately started measuring the fluorescence intensity of the supernatant every 5 min at 37°C in an air-saturated condition. The time course of the fluorescence intensity after starting the maturation reaction was described by an exponential growth curve fairly well for both proteins (Fig. S3). We derived the half times at which the fluorescence intensity reached the half of the amplitude of the exponential growth, and the results were 14.6 ± 0.9 and 36.7 ± 4.5 min (mean ± standard error, *n* = 3) for Kohinoor2.0 and Kohinoor, respectively. Thus, the maturation speed of Kohinoor2.0 was 2.5-fold faster than that of Kohinoor, demonstrating that Kohinoor2.0 should be more useful than Kohinoor for assured fluorescent labeling with less defect in observation.

**Fig. S3.** Measurement of chromophore maturation of Kohinoor2.0 and Kohinoor. The fluorescence intensity was measured from a cell lysate in a quartz cuvette in an air-saturated and temperature-controlled condition. Time zero denote the time at which the fluorescence intensity measurement started on a fluorescence spectrophotometer. The temperature was 37°C.

**S4. Speed of photobleaching**

We compared photobleaching speed of Kohinoor2.0 and Kohinoor exited at 488 nm. We transfected COS7 with an expression vector pcDNA3 containing a gene of Kohinoor2.0 fused with vimentin, an intermediate filament protein, or a gene of Kohinoor fused with vimentin. Thereby, we observed vimentin intermediate filaments labeled with Kohinoor2.0 or Kohinoor in the COS7 cells at 37°C on an epi-fluorescence microscope with excitation at 488 nm and 1 W/cm2 (Fig S4a). We took time trajectories of fluorescence intensity from regions with a size of ~1.3 × ~1.3 μm (~50 × ~50 pixels) on the specimen plane, and typical time trajectories from Kohinoor2.0-vimentin and Kohinoor-vimentin are shown in Fig S4b. The data shows that the fluorescence intensity decreased with time because of photobleaching, but Kohinoor sometimes showed a time lag before the fluorescence decay started, whereas Kohinoor2.0 little showed such a time lag (Fig S4b). Because on-switching also occurs with irradiation at 488 nm, the time trajectory of Kohinoor suggests that Kohinoor in equilibrium at 37°C may contain a small fraction of the off state. Therefore, we used a time interval from 3 min to 30 min in the time trajectories from Kohinoor for the least squares fitting analysis with a single exponential decay to calculate the time constants of photobleaching, whereas a time interval from 0 min to 30 min for Kohinoor2.0 (Fig. S4b). As shown in Table S2, the photobleaching rate constant of Kohinoor2.0 was 1.3-fold higher than that of Kohinoor. However, Kohinoor2.0 showed a 1.8-fold lower photobleaching quantum yield, meaning that the probability of photobleaching per excitation/de-excitation cycle is lower for Kohinoor2.0 than Kohinoor.


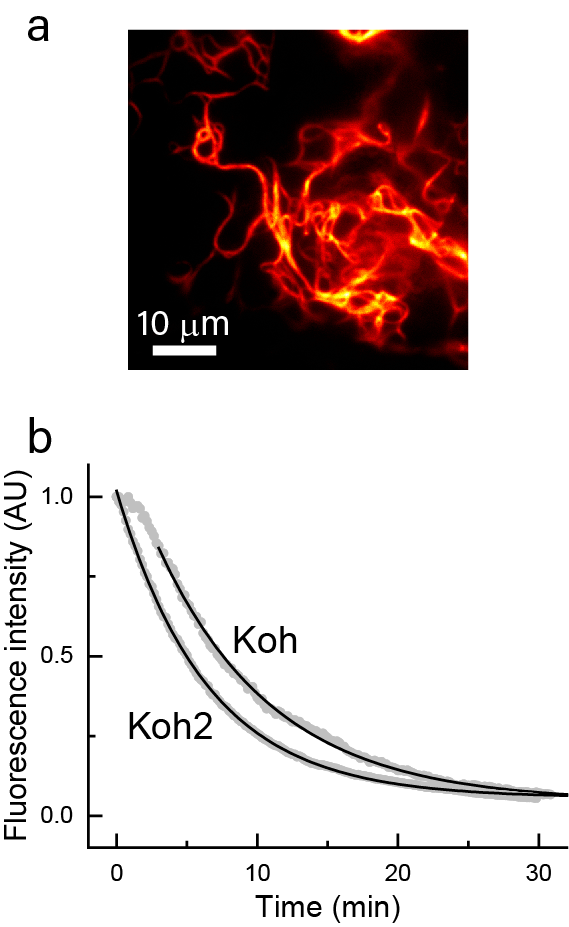


**Fig. S4.** Photobleaching measurement of Kohinoor2.0 and Kohinoor in COS7 cells. (a) A fluorescence image of Kohinoor2.0-vimentin intermediate filaments in a COS7 cell. (b) Typical time trajectories of fluorescence intensity taken from Kohinoor2.0- and Kohinoor-vimentin intermediate filaments in COS7 cells. The data points from the measurement are shown in gray dots. The solid curves are single exponential decay functions. The cells were irradiated with a light at 488 nm and 1 W/cm2. Temperature: 37°C. Koh2: Kohinor2.0. Koh: Kohinoor.

**Table S2.** Parameters of photobleaching

|  | Absorption cross section  (×1017 cm2;  On state, 488 nm)* |  | Photobleaching  (488 nm, 1 W/cm2) | |
| --- | --- | --- | --- | --- |
|  |  |  | Rate constant  (min−1)† | Photobleaching quantum yield  ×105‡ |
| Kohinoor2.0 | 10.9 ± 0.4 |  | 0.157 ± 0.007 | 1.16 ± 0.06 |
| Kohinoor | 3.79 ± 0.11 |  | 0.118 ± 0.006 | 2.12 ± 0.12 |

*mean ± SE; *n* = 3.

†mean ± SE; *n* = 27 and 21 for Kohinoor2.0 and Kohinoor, respectively.

‡The standard errors were calculated by error propagation [5].

**S5. Analysis of the pH profile of Kohinoor2.0 and Kohinoor**

We employed a regularized maximum likelihood estimation technique to perform the fitting of *A*model (Eq. (4)) with the observed absorbance of Kohinoor2.0 and Kohinoor (Fig. 2c). Because three conformation states with distinct absorption spectra were likely to be involved in the pH-dependent behavior of the chromophore phenolic group (Fig. 2a,b), we devised a three-state model (Fig. 2e). However, the three-state model led to the need to estimate a fairly substantial number of parameters by least squares fitting calculation, and unfortunately, the values of *A*1,N , *A*2,N, and *A*3,N in Eq. (4) computed by the nonlinear least squares fitting with the Levenberg-Marquardt method did not show fine convergence. Therein, we were aware that GFP-like proteins with the neutral chromophore phenolate exhibits very low absorbance at 498 nm [10]. Therefore, we assumed that the absorbance of the M1·H+ and M2H·H2+ was close to zero, and used a term of (*A*1,N2 + *A*2,N2) as the regularizer to perform the maximum likelihood estimation with the Tikhonov regularization [11]. To perform this calculation, we devised a loss function given by

(S5)

where *xi* is the *i*-th point of [H+] in the measurement, *Ai* is an absorbance measured at [H+]=*xi*, and *λ* is an adjustable parameter which controls the effect of the regularization term. We typically used a value around 10−4 for *λ*. In the estimation calculation, we sought the parameter values of *A*1,A, *A*2,A, *A*3,A, *A*1,N, *A*2,N, *A*3,N, *L*1,H, *L*2,H, *K*1, *K*2, and *K*3 such that the loss function *G* was minimized. We performed this regularized maximum likelihood estimation by using the FindFit module in the platform of Mathematica software (Version 12.0, Wolfram, Champaign, IL, USA). Additionally, we also analyzed the pH profile of Kohinoor (Fig. 2c) with the same three-state model (Fig. 2e). In this case, we used a term of (*A*1,N2 + *A*2,N2 + *A*3,N2) as the regularizer to perform the regularized maximum likelihood estimation, because the absorbance at 498 nm seemed to approach to zero as the pH decreased (Fig. 2c). Thus, in the case of Kohinoor, we used a loss function of *G′* given by,

(S6)

where we typically used a value around 10−4 for *λ*.

**S6. pH-dependent mole fractions of the molecular species**

Mole fractions of M1, M2H+, M3H22+, M1•H+, M2H•H2+, and M3H2•H3+ were calculated by the three-state model (Eqs. (1–3), Fig. 2e) with the parameter values derived from the least squares fitting (Table 2). The mole fractions were computed as follows

, ,

, ,

, , (S7)

where *P* is a binding polynomial (Eq. (3)). These mole fractions as a function of pH are shown in Fig. S5. Furthermore, the mole fractions of the anionic and neutral chromophore were computed by, respectively,

, (S8)

and

. (S9)

For the mole fractions of the anionic and neutral chromophore, see Fig. 2d.

**Fig. S5.** pH dependence of the mole fractions of the molecular species for Kohinoor2.0 and Kohinoor as computed by Eq. (S7) and parameter values in Table 2. (a) Mole fractions of M3H22+, and M3H2•H3+. (b) Mole fractions of M2H+, and M2H•H2+. (c) Mole fractions of M1 and M1•H+. Note that dark curves correspond to the conformation states with the neutral chromophore phenol, and red curves to those with the anionic chromophore phenolate.

**S7. pH-Dependence of fluorescence emission**

We measured pH-dependent fluorescence emission of Kohinoor2.0 and Kohinoor in the on state at 25°C. The fluorescence emission spectra of Kohinoor2.0 showed that the fluorescence intensity was significantly dependent on pH and the emission peak wavelength also changed with pH to some extent (516 nm at pH 5.0; 514 nm at pH 11.0) (Fig. S6a). Kohinoor2.0 showed higher fluorescence at 516 nm than Kohinoor below pH 9, but showed lower fluorescence than Kohinoor above pH 9. The pH-dependence of the fluorescence emission should relate to the protonation states of the chromophore and its surrounding amino acid residues. Although we analyzed the pH profile of absorbance with the three-state model (Fig. 2c,e, Eq. (4)), one of the simplest reaction schemes that fit the fluorescence-pH profile was a two-state model as follows:

| 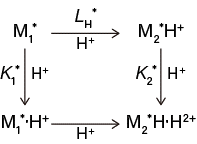 | (S10) |
| --- | --- |

where the M1* and M2*H+ states are fluorescently-bright states with the chromophore phenolate and M1*•H+ and M2*H•H2+ are fluorescently-dark states with the chromophore phenol (solid curves in Fig. S6b). Taking into consideration the Franck-Condon principle and the Kasha’s rule [12], the chromophore absorbance of a fluorescent protein should mainly reflect the light absorption of the ground state of the chromophore, and the fluorescence intensity should mainly reflect the deactivation of the lowest excited singlet state in addition to the populations of the conformation states in the ground state. The excitation of the chromophore is generally followed by the relaxation of the configuration of the chromophore and the surrounding amino acids [13], presumably leading to re-organization of the hydrogen-bond network inside the β-barrel. This could be responsible for the pH-dependent behavior of fluorescence different from that of absorbance.

**Fig. S6.** pH-dependence of fluorescence of Kohinoor2.0 and Kohinoor. (a) Fluorescence emission spectra of Kohinoor2.0 between pH 5.0 and 11.0. (b) pH-profiles of fluorescence intensity at 516 nm taken from Kohinoor2.0 and Kohinoor. The solid curves represent model functions derived from a reaction scheme in Eq. (S10). The values of p*K*1*, p*K*2*, and p*L*H* (Eq. (S10)) derived from the analysis were 9.2, 6.1, and 9.4, respectively, for Kohinoor2.0, and 9.1, 7.1, and 9.0, respectively, for Kohinoor. Excitation wavelength: 488 nm. Temperature: 25°C.

**S8. pH-dependence of photoswitching rates**

We measured photoswitching rates of purified Kohinoor2.0 and Kohinoor at various pH. We constructed an instrument for this measurement, in which laser beams at 488 nm and 405 nm were incident on a micro-cuvette (window size, 2.0 × 2.5 mm; light path length, 10.0 mm; T-703M-ES-10.50A; Tosoh Quartz, Tokyo, Japan) containing a Kohinoor2.0 or Kohinoor solution at 25°C (Fig. S7a). Time trajectories of their fluorescence was measured by a photosensor module (H10721-20; Hamamatsu Photonics, Hamamatsu, Japan) combined with an amplifier (C11184; Hamamatsu Photonics) and a data acquisition board (USB-1602HS-2AO; Measurement Computing, Norton, MA, USA).

In the measurement, we irradiated a sample of Kohinoor2.0 or Kohinoor with an off-switching light at a power density of ~20 mW/cm2 and a wavelength of 405 nm for 1 min, and then, we subsequently irradiated the sample with an excitation/on-switching light at ~17 mW/cm2 and 488 nm to take the time course of the fluorescence increase during the on-switching (Fig. S7b). After the fluorescence intensity reached a plateau, we further irradiated the sample with the excitation/on-switching light for additional 30 s, and then, we added the off-switching light at ~20 mW/cm2 and 405 nm to follow a relaxation toward an equilibrium between the on- and off-states (Fig. S7c). As shown in Fig. S7b,c, the time trajectories were well-described by single exponential functions, and thus, we computed the rate constants of on- and off-switching from the time constants of the on-switching and relaxation processes according to our previous report [1]. The measured photoswitching rates were normalized by the power density of the irradiation lights, which we measured every time just before the measurement.

Figure S7d shows pH-profiles of on-switching rates for Kohinoor2.0 and Kohinoor. The on-switching rates of both proteins showed a plateau below pH 7, but the rates increased as the pH increased above pH 7. The absorption spectrum of the off state (Fig. 1b) indicates that the anionic form of chromophore phenolate (in the trans configuration) should be largely dominant, and therefore, only a small fraction of the proteins can further convert into the anionic form if the pH is increased from 7 to 11. Hence, the several-fold enhancement of the on-switching rate from pH 7 to 11 suggests that amino acid residues surrounding the chromophore which deprotonate above pH 7 may have an effect on the structural freedom of the excited chromophore to promote its isomerization.

The pH-dependence of the off-switching rate seemed to be divided into three regimes (pH < 6, pH 6–8, and pH > 8), and the off-switching was fast in the range of pH6–8 (Fig. S7e). Note that the off-switching occurs, when the cis chromophore with the neutral phenol is excited, i.e., the chromophore of Kohinoor2.0 or Kohinoor in the M1•H+, M2H•H2+, or M3H2•H3+ state (Fig. 2e) is excited. According to Fig. S7f, the pH ranges of the populations of these conformational states seem to be consistent with the pH regimes of the off-switching rates. Based upon this speculation, the off-switching rates of the M1•H+ and M2H•H2+ and M3H2•H3+ states were crudely estimated to be 100, 30, and 18 (s−1 W−1 cm2), respectively, as calculated by *k*Off/*f*, where *f* is mole fractions of the M1•H+ and M2H•H2+ and M3H2•H3+ states at pH 10.5, 7.5, and 5.0, respectively (Fig. S7e,f). This result suggests that, like the on-switching, the deprotonated amino acid residues surrounding the chromophore may have an effect on the flexibility of the excited chromophore to promote its isomerization. However, further investigation should be necessary to reveal the interplay of the chromophore and surrounding amino acids in the molecular mechanisms of the on-switching and off-switching.


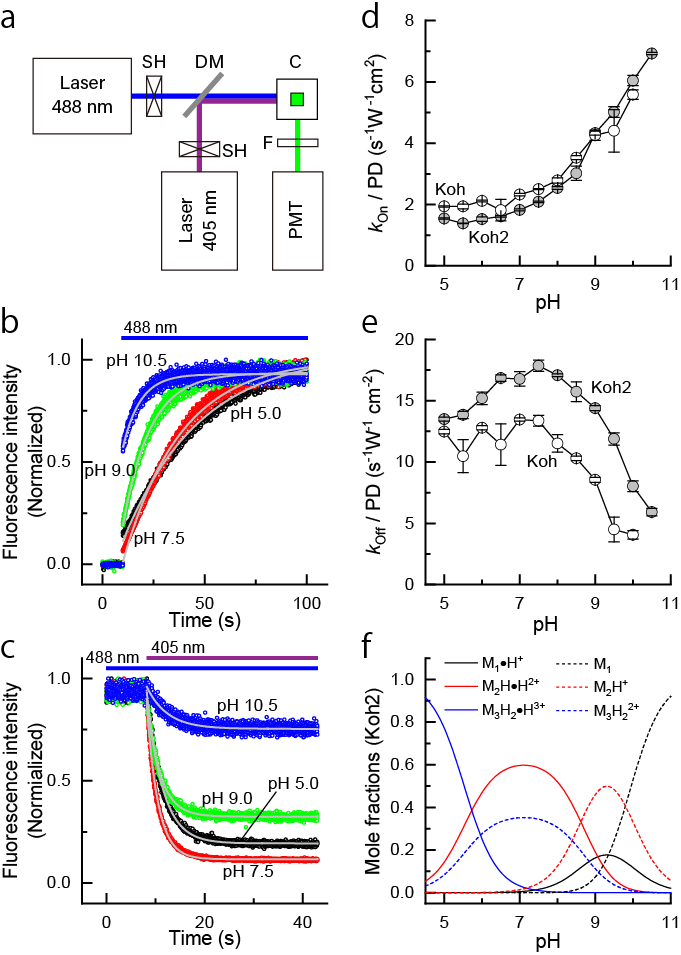


**Fig. S7.** pH-dependence of photoswitching rates of purified Kohinoor2.0 and Kohinoor at 25°C. (a) A schematic diagram of a photoswitching measurement instrument. C: cuvette; DM: dichroic mirror; F: bandpass filter; PMT: photomultiplier tube; SH: electronic shutter. (b) Time trajectories of fluorescence intensity in the conversion from the off state to the on state with the irradiation of the on/excitation light. The line above the graph indicates the timing of the light irradiation at 488 nm. (c) Time trajectories of fluorescence intensity in the conversion from the on state to an equilibrium with the irradiation of the on/excitation light and the off-switching light. The lines above the graph indicate the timings of the light irradiation. (d) pH profiles of the on-switching rate constants normalized to 1 W/cm2 for Kohinoor2.0 and Kohinoor (*n* = 3). PD: power density. (e) pH profiles of the off-switching rate constants normalized to 1 W/cm2 for Kohinoor2.0 and Kohinoor (*n* = 3). (f) pH-dependent mole fractions of the conformation states: M1, M1•H+, M2H+, M2H•H2+, M3H22+, and M3H2•H3+. The data were calculated according to Section S6

**S9. Localization of Kohinoor2.0-fused proteins and polypeptides in HeLa cells**

We examined the localization of Kohinoor2.0 fused to several proteins and a polypeptide in HeLa cells. Figure S8b–i shows the typical results of the localization of histone 2B, a targeting sequence of subunit-VIII precursor of human cytochrome c oxidase (tsCOX8), β-tubulin, LifeAct, vimentin, clathrin, fibrillarin, and zyxin, as observed by a confocal microscope. The data demonstrated that the fused Kohinoor2.0 exhibits in-cell localization consistent with previous observations [1,6,9]. In fact, Kohinoor2.0 fused with histone 2B was observed to localize in the nucleus (Fig. S8b), while Kohinoor2.0 fused with β-tubulin, LifeAct, and vimentin showed fluorescently-labeled microtubules, actin filaments, and intermediate filaments, respectively (Fig. S8d, e, and f, respectively). Thus, the data demonstrated that the localization of these proteins and polypeptides is not affected by conjugation with Kohinoor2.0. Furthermore, we also observed unfused Kohinoor2.0 expressed in HeLa cells, which dispersed over a cell without noticeable aggregation (Fig. S8a).


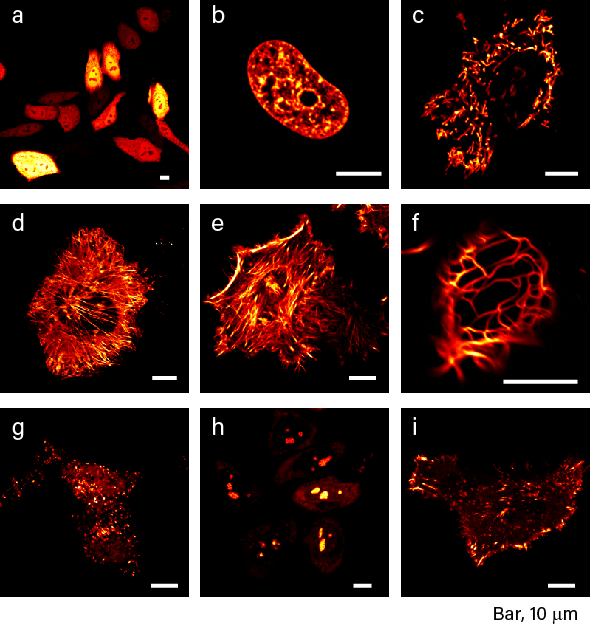


**Fig. S8.** Confocal observation of the localization of Kohinoor fused to proteins and polypeptide in live HeLa cells. (a) Unfused Kohinoor2.0 in cytoplasm. (b) Histone 2B in nucleus. (c) tsCOX8 in mitochondria. (d) β-tubulin in microtubules. (e) LifeAct in actin filaments. (f) Vimentin in intermediate filaments. (g) Clathrin spots. (h) Fibrilarin in nucleolus. (i) Zyxin in focal adhesions. Observation was conducted using an FV1000 confocal microscope at room temperature.

**S10. Spatial resolution in SPoD-OnSPAN imaging of Kohinoor2.0 and Kohinoor**

We examined spatial resolutions of SPoD-OnSPAN images with Kohinoor2.0 and Kohinoor. We performed SPoD-OnSPAN observation of actin filaments labeled with LifeAct-Kohinoor2.0 or LifeAct-Kohinoor expressed in COS7 cells, and then reconstructed super-resolved images (Fig. S9a,b). We took line profiles of actin filaments in the super-resolved images to measure full widths at half maximum (FWHM) by least squares fitting with a Gaussian distribution function (Fig. S9c–f). The FWHM was measured to be typically 50–70 nm, and clear difference in FWHM was not noticeable between Kohinoor2.0 and Kohinoor. In our previous study [1], we reported FWHM values of 70–80 nm, and thus, the present results in Fig. S9e,f were slightly better. This would be because we used a higher pixel resolution of 20 nm/pixel in the present observation, whereas we used a lower pixel resolution of 40 nm/pixel in the observation of Fig. 3 in ref [1].


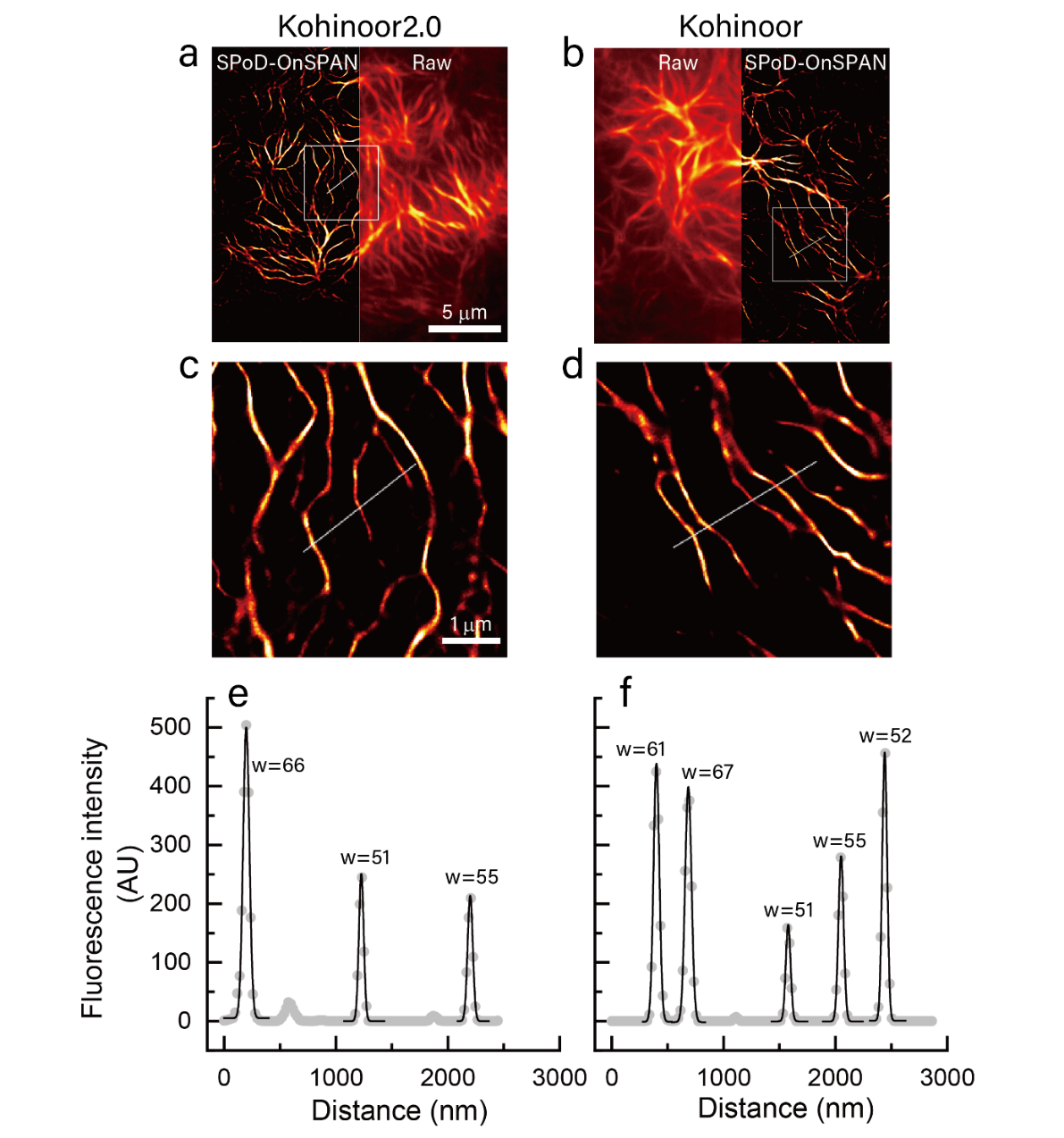


**Fig. S9.** Typical spatial resolution in SPoD-OnSPAN observation with Kohinoor2.0 and Kohinoor. (a,b) Wide-field and SPoD-OnSPAN images of actin filaments labeled with (a) LifeAct-Kohinoor2.0 and (b) LifeAct-Kohinoor expressed in a COS7 cell. (c,d) Super-resolved images in an expanded scale taken from regions enclosed by squares shown in panels (a) and (b), respectively. (e,f) Line-profiles of fluorescence intensity along lines shown in panels (c) and (d), respectively. The solid curves are Gaussian distribution functions fitted to the data points. FWHM (nm) of the peaks are shown in the panels. The illumination power densities were 0.99 W/cm2 and 0.67 W/cm2 for 488 nm and 405 nm, respectively.

**S11. List of supplementary image data**

We provide time-lapse SPoD-OnSPAN images of actin filaments labeled with LifeAct-Kohinoor2.0 and mitochondria labeled with tsCOX8-Kohinoor2.0 in COS7 cells shown in Fig. 4. Files of “*-RawAvg-*.tif” are image stacks, each frame of which is the average of 18 or 9 raw images. Files of “*-SPoD-OnSPAN-*.tif” are image stacks of reconstructed super-resolved images. For the detail of experimental condition, see Fig. 4 and the text. Table S3 shows some parameters for the time-lapse images. Image J software is useful to play the multiple TIFF movies.

**Table S3.** Parameters for the supplementary movies of actin filaments and mitochondria.

| Files | Fig4a-*.tif | Fig4c-*.tif |
| --- | --- | --- |
| Labeling | LifeAct-Kohinoor2.0 | tsCOX8-Kohinoor2.0 |
| Frame rate | 0.33 frames/min | 2 frames/s |
| Number of raw frames used for the reconstruction of one super-resolved image | 18 frames | 9 frames |
| Scale factor | 20 nm/pixel | 20 nm/pixel |

**REFERENCES**

1. Wazawa T, Arai Y, Kawahara Y, Takauchi H, Washio T, and Nagai T (2018) Highly biocompatible super-resolution fluorescence imaging using the fast photoswitching fluorescent protein Kohinoor and SPoD-ExPAN with *Lp*-regularized image reconstruction. *Microscopy (Oxf)* **67**, 89–98.
2. Cantor C R, and Schimmel P R (1980) Biophysical Chemistry, part II: techniques for the study of biological structure and function. (W. H. Freeman, San Francisco.)
3. Peterman E J G, Brasselet S, and Moerner W E (1999) The Fluorescence Dynamics of Single Molecules of Green Fluorescent Protein. *J. Phys. Chem. A* **103**, 10553–10560.
4. Shinoda H, Lu K, Nakashima R, Wazawa T, Noguchi K, Matsuda T, and Nagai T (2019) Acid-tolerant reversibly switchable green fluorescent protein for super-resolution imaging under acidic conditions. *Cell Chem. Biol.* **26**, 1469–1479.
5. Bevington, P R, and Robinson D K (1992) Data reduction and error analysis for the physical sciences, 2nd Ed. (McGraw-Hill, New York)
6. Tiwari D K, Arai Y, Yamanaka M, Matsuda T, Agetsuma M, Nakano M, Fujita K, and Nagai T (2015) A fast- and positively photoswitchable fluorescent protein for ultralow-laser-power RESOLFT nanoscopy. *Nat. Methods* **12**, 515–518.
7. Nienhaus K, and Nienhaus G U (2016) Chromophore photophysics and dynamics in fluorescent proteins of the GFP family. *J. Phys. Condens. Matter* **28**, 443001.
8. Reid B G, and Flynn G C (1997) Chromophore formation in green fluorescent protein. *Biochemistry* **36**, 6786–6791.
9. Shinoda H, Ma Y, Nakashima R, Sakurai K, Matsuda T, and Nagai T (2018) Acid-Tolerant Monomeric GFP from *Olindias formosa*. *Cell Chem. Biol.* **25**, 330–338.
10. Tsien R Y (1998) The green fluorescent protein. *Annu. Rev. Biochem.* **67**, 509–544.
11. Bishop C M (2006) Pattern recognition and machine learning. (Springer, New York.)
12. Turro N J (1991) Modern molecular photochemistry. (University Science books, Sausalito)
13. Lakowicz, J R (2006) Principles of fluorescence spectroscopy, 3rd ed. (Springer, New York)
