# Supplementary figures and images for: A photoswitchable fluorescent protein for hours-time-lapse and sub-second-resolved super-resolution imaging
Source: Microscopy (Oxf). 2021 Jan 22;70(4):340–52. doi: 10.1093/jmicro/dfab001 (PMC8350982; doi:10.1093/jmicro/dfab001)

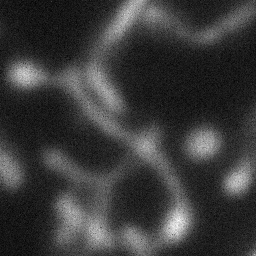

Supplement: dfab001_Supp [file dfab001_supp.zip › Fig4c-RawAvg-Mt/Fig4c-RawAvg-Mt.tif]
